# RAD51AP1 is a versatile RAD51 modulator

**DOI:** 10.1073/pnas.2514728122

**Published:** 2025-12-03

**Authors:** Lucas Kuhlen, Bilge Argunhan, Pengtao Liang, Janet Zhong, Laura Masino, Xiaodong Zhang

**Affiliations:** ^a^Section of Structural and Synthetic Biology, Faculty of Medicine, Imperial College, London SW7 2AZ, United Kingdom; ^b^DNA Processing Machines Laboratory, Francis Crick Institute, London NW1 1AT, United Kingdom; ^c^Structural Biology Science Technology Platform, Francis Crick Institute, London NW1 1AT, United Kingdom

**Keywords:** RAD51 recombinase, RAD51AP1, filaments modulation, homologous recombination, structural biology

## Abstract

RAD51AP1 is an emergent key factor in homologous recombination (HR), the major pathway for accurate repair of DNA double-strand breaks, and in alternative lengthening of telomeres (ALT). Depletion of RAD51AP1 diminishes HR and overexpression is common in cancer, where it is associated with malignancy. Here, we show that RAD51AP1 has a hitherto unknown role in modulating the RAD51 recombinase, the central player in HR. Through a combination of biochemistry and structural biology, we reveal that RAD51AP1 possesses at least three RAD51-binding sites that facilitate its binding across two adjacent RAD51 molecules. We uncover a previously unidentified RAD51-binding mode that stabilizes the RAD51 N-terminal domain and protomer interface in the filaments. We uncover a previously undescribed role for RAD51AP1 in stabilizing RAD51-ssDNA filaments and promoting strand exchange. Our structural data provide the molecular basis for how RAD51AP1 binding induces conformational changes that promote RAD51 DNA association and oligomerization, therefore promoting filament nucleation, stabilization, and strand exchange. Further, we resolved structures of RAD51-ssDNA filaments in the presence of Mg^2+^-ATP and upon hydrolysis to Mg^2+^-ADP, revealing that RAD51 filaments expand upon ATP hydrolysis and explaining how ADP reduces RAD51–DNA binding. Our findings reveal RAD51AP1 as a versatile RAD51 modulator and RAD51 filament remodeler and shed previously unidentified insights into the modulation of HR, which is critical for the maintenance of genome stability.

## Significance

RAD51AP1 is an emergent key factor in homologous recombination (HR), the major pathway for accurate repair of DNA double-strand breaks, and in alternative lengthening of telomeres (ALT). Depletion of RAD51AP1 diminishes HR and overexpression is common in cancer, where it is associated with malignancy. We show that RAD51AP1 serves as a versatile modulator of the RAD51 recombinase, the central player in HR. Our study uncovers a previously unidentified RAD51-binding mode and characterizes a hitherto overlooked role for RAD51AP1 in stabilizing RAD51-ssDNA filaments and promoting strand exchange. Our findings provide mechanistic insights into RAD51 recombinase and RAD51AP1.

The human genome is constantly being damaged and repaired. DNA double-strand breaks (DSBs) constitute the most cytotoxic form of DNA damage, and as a result, their faithful repair is critical for the maintenance of genome stability. Homologous recombination (HR) is the major pathway for high-fidelity repair of DSBs (1, 2). Disruption of HR leads to genomic instability, which is a strong driver of tumorigenesis (3). Furthermore, HR-directed repair is one of the major pathways to resolve stalled or damaged replication forks and for alternative lengthening of telomeres (ALT) (4–6).

The central protein in HR is the RAD51 recombinase. RAD51 forms a nucleoprotein filament with single-stranded DNA (ssDNA) generated from enzymatic end resection at the DSB site (7, 8). The RAD51-ssDNA filament performs the recombinase function through searching and identifying homologous sequences in intact donor double-stranded DNA (dsDNA), invading the dsDNA to allow the pairing of the ssDNA with the complementary strand from donor DNA, and using it as a template for DNA synthesis, resulting in repair (9, 10). However, unlike its bacterial orthologue RecA, the intrinsic recombinase activity of RAD51 is low, and eukaryotic HR requires a multitude of auxiliary factors to promote RAD51 activities for proficient DSB repair (11). Furthermore, erroneous HR can lead to unregulated strand invasion and chromosome instability (12, 13), and chromosomal accumulation of RAD51 can lead to cell toxicity (13, 14). RAD51’s activity thus needs to be tightly regulated. One emergent RAD51 modulator is RAD51AP1 (RAD51 associated protein 1), a protein that directly binds to RAD51 (15) and promotes its activities in HR, thus contributing to the maintenance of genome stability (16, 17). Importantly, RAD51AP1 is overexpressed in many types of cancer, including breast and ovarian cancer, and this overexpression is associated with a poor prognosis (18). RAD51AP1 deletion improves survival in mouse models of breast cancer and its expression is associated with cancer stem cell self-renewal, which is highly dependent on functional DNA repair (19), confirming its important roles in HR and cancer development. More recently, RAD51AP1 has been shown to play key roles in R-loop formation and ALT (20–22).

**Fig. 1.**
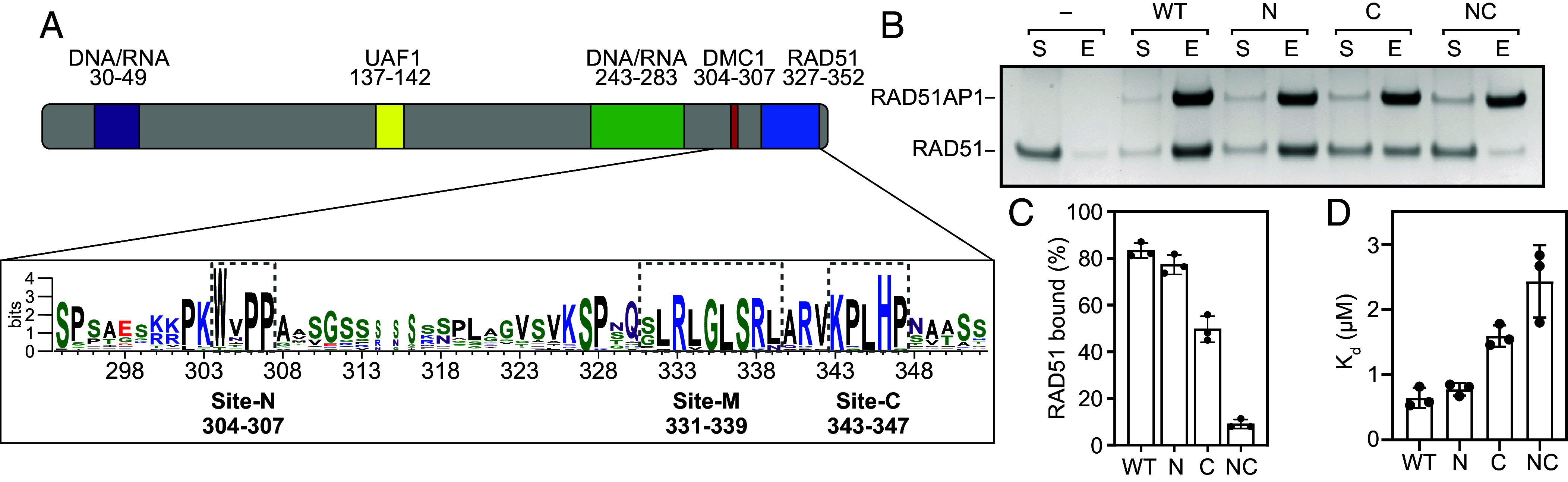
RAD51AP1 binds to RAD51 via two distinct binding sites. (*A*) Schematic of RAD51AP1 showing regions of interest. (*B*) Pull-down experiment of full-length RAD51AP1 (6xHis-tagged wild type or mutants) and RAD51 showing supernatant (S) and eluate (E). (*C*) Quantification of (*B*). Averages are shown with error bars depicting SD (n = 3). (*D*) K_d_ values for the binding of full-length RAD51AP1 and RAD51, determined by biolayer interferometry (BLI). Averages are shown with error bars depicting SD (n = 3). *P*-values for comparison of WT with Site-C and Site-NC mutants were <0.0001, WT with Site-C was 0.3044, Site-N with Site-C was 0.0001 and Site-NC and Site-N and Site-C was <0.0001. Statistical test was one-way ANOVA followed by Tukey’s correction for multiple comparisons.

Sequence and secondary structure analysis as well as biochemical investigation (17) suggest that RAD51AP1 is a largely intrinsically disordered protein (Fig. 1*A* and *SI Appendix*, Fig. S1*A*). Earlier biochemical and genetic studies identified a RAD51-binding site and two DNA/RNA binding sites (Fig. 1*A*), which are all required for full function in cells (16, 20, 22). In HR, it is thought that RAD51AP1 engages with RAD51 through its C-terminal RAD51-binding site (16, 23) and then captures dsDNA with its DNA binding sites to form a molecular bridge between the filament and the strand exchange donor (16, 17). In ALT, RAD51AP1 has been proposed to bind to telomere-specific RNA (TERRA) and promote R-loop formation, which subsequently promotes D-loop formation and HR-directed repair (6, 20, 21); it is unclear if RAD51-binding activities also play a role here. RAD51AP1 has also been shown to bind to nucleosomes, supporting the notion that it can promote the pairing of a DSB site with the sister chromatid in cells (24).

Despite its importance in HR and other processes that involve RAD51, the exact molecular mechanism of RAD51AP1 in these activities is unknown. Importantly, thus far, due to the instability of RAD51–DNA filaments in the presence of Mg^2+^ and ATP, current structures of RAD51–DNA filaments have been resolved either by replacing Mg^2+^ with Ca^2+^, which curbs ATP hydrolysis (25), or using the nonhydrolyzable ATP analogue AMP-PNP (26) or the hydrolysis deficient RAD51-K133R mutant (27–30). Single-molecule analysis has suggested that ADP-bound protomers dissociate from the filament (31), providing a feasible explanation for why restricting ATP hydrolysis may lead to enhanced filament stability.

Here, using cryogenic electron microscopy, we resolved structures of RAD51-ssDNA filaments in complex with Mg^2+^-ATP and Mg^2+^-ADP, revealing conformational changes upon ATP hydrolysis and the structural basis for their distinct DNA binding properties. Furthermore, we obtained structures of RAD51AP1 in complex with RAD51-ssDNA filaments, uncovering the mechanism of RAD51AP1 in RAD51 filament modulation. Combined with biochemical studies and mutagenesis, we show that RAD51AP1 possesses three RAD51-binding sites that facilitate its binding across two adjacent RAD51 molecules. We uncover a previously unidentified RAD51-binding mode, as well as characterize a hitherto overlooked role for RAD51AP1 in stabilizing RAD51-ssDNA filaments and reordering structural elements in RAD51 that promote strand exchange. Our studies reveal that RAD51AP1 acts as a RAD51 modulator via strengthening protomer–protomer interactions, promoting RAD51 oligomerization and reordering its DNA binding sites, thereby facilitating filament nucleation, stabilization, and strand exchange.

## Results

### RAD51AP1 Binds to RAD51 Via Distinct Sites.

Previous biochemical analyses identified a RAD51-binding site near the extreme C terminus of RAD51AP1, with the H346A mutation severely impairing RAD51 binding (16). Upon examination of the RAD51AP1 sequence, we noticed a stretch of conserved residues upstream of this binding site (Fig. 1*A* and *SI Appendix*, Fig. S1*B*). Interestingly, these residues were previously shown to comprise the binding site for DMC1, the meiosis-specific RecA-family recombinase in eukaryotes (32). We refer to this DMC1 binding site as Site-N, and the known RAD51-binding site as Site-C for their relative positions in the sequence. Given the structural and sequence similarity between RAD51 and DMC1, we hypothesized that Site-N may also be involved in RAD51 binding. To test this, we mutated five conserved residues (K300, K303, W304, P306, and P307) in Site-N to alanine. We also included the known mutant (RAD51AP1-H346A) that disrupts RAD51 binding in our analysis as a Site-C mutant, and designed a RAD51AP1 variant in which both sites are mutated (referred to as NC). Along with RAD51, these RAD51AP1 variants were purified to homogeneity (*SI Appendix*, Fig. S1*C*).

As expected, RAD51 can be pulled down by RAD51AP1 and the Site-C mutant showed a reduction in binding (Fig. 1 *B* and *C*). Compared with wild-type RAD51AP1, the Site-N mutant did not show an appreciable reduction in RAD51 binding. Interestingly, when both binding sites were mutated, the binding was almost abolished (Fig. 1 *B* and *C*). To quantify these interactions, BLI experiments were conducted and the results are consistent with those of pull-down experiments (Fig. 1*D* and *SI Appendix*, Fig. S1*D*). Wild-type protein has a dissociation constant of ~0.6 μM, which is comparable to the Site-N mutant, whereas the Site-C mutant decreased the binding affinity by ~2.5-fold, and the Site-NC double mutant showed a ~fourfold reduction in affinity (Fig. 1*D*). These results indicate that RAD51AP1 binds to RAD51 via at least two distinct sites, and these two sites synergistically enhance the binding, although Site-C is the major binding site.

### RAD51AP1 Promotes RAD51 Filament Nucleation, Stabilizes Filaments, and Stimulates Strand Exchange.

**Fig. 2.**
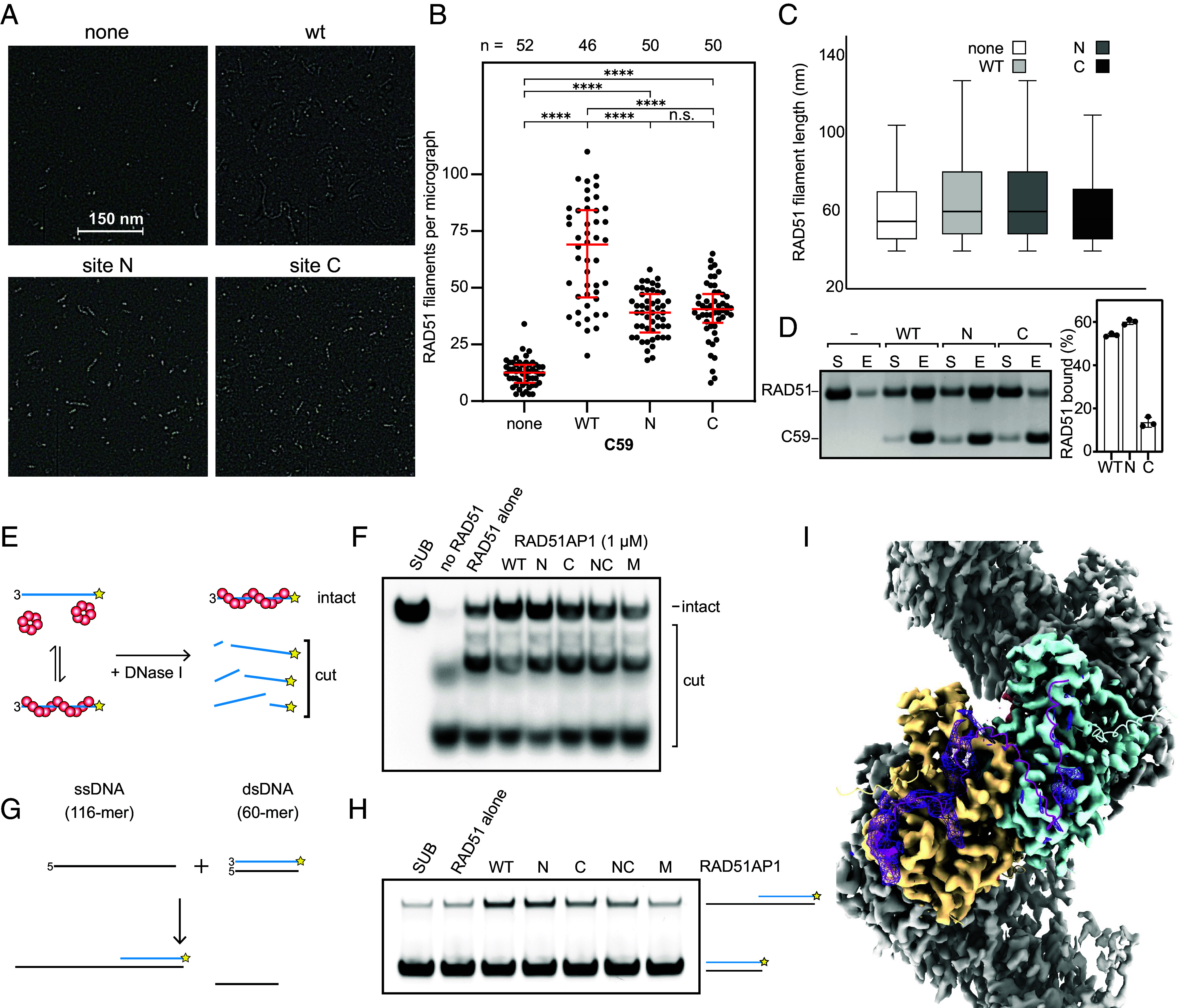
RAD51AP1 promotes filament formation, stability, and strand exchange. (*A*) Representative negative-stain electron micrographs of RAD51 filaments. RAD51 filaments were prepared either with RAD51 alone (none) or with C59 (WT or mutants) added. (*B*) Quantification of RAD51 filaments per micrograph. The red bar represents the median with lower and upper quartiles. (*C*) Box plot quantification of RAD51 filament length showing upper and lower quartiles. Line = median, whiskers = 1.5 × Interquartile range. none, n = 642; WT, n = 2,997; N, n = 1,918; C, n = 1,993. (*D*) Pull-down experiment of C59 (6xHis-tagged wild type or mutants) and RAD51 showing supernatant (S) and eluate (E). For quantification, averages are shown with error bars depicting SD (n = 3). *P*-values for comparisons of WT with Site-N was 0.0048, and Site-C with WT or Site-N were <0.0001. Statistical test was one-way ANOVA followed by Tukey’s correction for multiple comparisons. (*E*) Schematic of nuclease protection assay to assess RAD51 filament stability. The star represents fluorophore (Cy5). (*F*) Representative gel image showing RAD51 filament stabilization by full-length RAD51AP1 (wild type or mutants). (*G*) Schematic of oligonucleotide strand exchange assay. The star represents fluorophore (Cy5). (*H*) Representative gel image showing the stimulation of RAD51-driven strand exchange by full-length RAD51AP1 (wild type or mutants). (*I*) CryoEM map of the Ca^2+^-ATP bound RAD51-ssDNA filament in the presence of RAD51AP1. with an AlphaFold3 model of the RAD51AP1 C terminus (C59, magenta) bound to two RAD51 (yellow and blue), RAD51AP1 (magenta). Additional density to those of RAD51 is shown as a mesh.

Given that RAD51AP1 is known to promote HR via RAD51, we next tested whether it can stimulate RAD51 activities including filament nucleation, stabilization, and strand exchange. To directly assess the effects of RAD51AP1 on RAD51-ssDNA filaments, we used negative stain electron microscopy to visualize and quantify the RAD51 filaments formed in the presence of Mg^2+^-ATP (Fig. 2 *A*–*C*). Because the addition of RAD51AP1 to filaments caused aggregation, we employed a C-terminal fragment that contains both binding sites (C59, similar to C60 used in ref. 32) that did not cause aggregation and binds to RAD51 in a similar fashion as the full-length protein (Figs. 1*B* and 2*D*). Similar to previous studies of other RAD51 modulators such as BRCA2 (33) and fission yeast Swi5-Sfr1 (34), addition of C59 led to an increase in the number of filaments observed (Fig. 2*B*) and shifted the distribution of filament lengths toward longer filaments (Fig. 2*C*), suggesting it increases filament nucleation and stability. Mutating Site-C impacted both filament numbers and filament length, consistent with it being a major binding site (Fig. 2 *B* and *C*). Interestingly, mutation of Site-N caused a reduction in filament number (Fig. 2*B*) but not length (Fig. 2*C*) even though the mutation did not reduce binding affinity significantly (Fig. 2*D*). These results suggest that site-N mainly plays a role in nucleation.

To corroborate these findings in the context of full-length proteins, we next tested the ability of full-length RAD51AP1 to stabilize RAD51 filaments in a nuclease protection assay (14, 35). When RAD51 forms a filament around fluorescently labeled ssDNA, the ssDNA is protected from nucleolytic digestion; more stable filaments are more resistant to nuclease digestion (Fig. 2*E*). Under our experimental conditions, RAD51 alone offered little protection against nuclease digestion (Fig. 2*F*). By contrast, when RAD51AP1 was added, ssDNA protection increased substantially. Mutating either site reduced the effects of nuclease protection, although Site-C mutation had a larger effect, consistent with the results of our negative stain electron microscopy experiments.

We next tested the effect of RAD51AP1 on strand exchange. We used a 60 bp double-stranded DNA with homology to a 116 nt ssDNA (Fig. 2*G*). Preincubating RAD51 with ssDNA allows for the formation of RAD51-ssDNA filaments, which can pair with homologous dsDNA, invade the duplex, then transfer strands to form a heteroduplex molecule that has 60 bp of dsDNA and 56 nt of ssDNA. Fluorescently labeling the complementary strand on the dsDNA allowed the detection of this product following electrophoresis (Fig. 2*G*). Indeed, under these experimental conditions, strand exchange was enhanced by RAD51AP1, with the Site-C and Site-N and Site-C double mutants displaying a noticeable defect in this assay (Fig. 2*H*).

### CryoEM Structure of the Ca^2+^-ATP RAD51-ssDNA Filament Bound to Full-Length RAD51AP1.

To understand how the two RAD51-binding sites of RAD51AP1 enhance RAD51 binding synergistically and how RAD51AP1 stabilizes RAD51-ssDNA filaments, we sought to determine the structure of RAD51AP1 in complex with RAD51-ssDNA filaments using cryoelectron microscopy.

Consistent with its binding across multiple RAD51 molecules, RAD51AP1 binding tends to induce filament clustering and bundling, preventing structural studies using single particle analysis. We therefore first attached RAD51-ssDNA filaments stabilized by Ca^2+^ to a carbon surface on EM grids, then incubated the grids with RAD51AP1 before flash freezing the grids, which resulted in well-separated filaments suitable for single particle analysis (*SI Appendix*, Fig. S2*A*). Image analysis and data processing led to a 3D reconstruction of the RAD51 filament at an overall resolution of 3.1 Å (*SI Appendix*, Fig. S2*A*). After selecting particles in which we observed extra density on the outside of the filament, we obtained a reconstruction of the filament at an overall resolution of 3.1 Å (Fig. 2*I* and Table 1 and *SI Appendix*, Fig. S2). The reconstruction has well resolved density for ssDNA bases, ATP bound in-between RAD51 monomers and side chains of RAD51, allowing us to build a structural model of RAD51-ssDNA filament (*SI Appendix*, Fig. S2 *B* and *C*). There is additional density on the surface of some of the RAD51 monomers that could correspond to RAD51AP1 (Fig. 2*I*). Using AlphaFold3 (36) to model a complex between a RAD51 dimer and C59 (*SI Appendix*, Fig. S2*D*), we obtained a structural model that allowed the residues 329 to 349 of RAD51AP1 to be fitted into the extra density (Fig. 2*I*).

**Table 1.** cryoEM data collection and model statistics

	RAD51 + RAD51AP1	RAD51	RAD51	RAD51 + RAD51AP1 (C29)
Nucleotide	Ca^2+^-ATP	Mg^2+^-ATP	Mg^2+^-ADP	Mg^2+^-ATP
Microscope	Titan Krios	Titan Krios	Titan Krios	Titan Krios
Voltage (keV)	300	300	300	300
Detector	K3 (Gatan)	K3 (Gatan)	K3 (Gatan)	K3 (Gatan)
Magnification	81,000	81,000	81,000	81,000
Defocus range (µm)	−3.0 to −0.9	−3.0 to −0.9	−3.0 to −0.9	−3.0 to −0.9
Frames/movie	40	50	50	50
Pixel size (Å)	1.08	1.08	1.08	1.08
Electron dose (e/Å^−2^)	42	53	53	53
Exposure (s)	3.85	4.6	4.6	4.6
Micrographs	5,001	3,096	3,096	3,233
Picked particles	2.4 million	5.6 million	5.6 million	6.1 million
Final particles	137,283	242,944	229,610	97,029
Processing method	Single particle analysis	Helical refinement	Helical refinement	Single particle analysis
Resolution (Å)	3.1	3.3	3.6	3.0
*Real-space refinement*				
Composition:				
Non-H atoms	15,950	14,932	14,791	16,097
Residues				
Protein	1,997	1,865	1,919	2015
Nucleotide	21	20	0	20
Ligands	21	19	18	17
Cross-correlation (CC, mask)	0.8268	0.8159	0.7813	0.7973
Bonds (rmsd)				
Lengths (Å)	0.0021	0.0022	0.0044	0.0025
Angles (°)	0.47	0.42	0.75	0.47
MolProbity score	1.28	1.24	1.68	1.29
Clash score	5.17	4.62	6.84	4.5
Rotamer outliers (%)	0.38	0.54	0.8	0.5
Ramachandran plot				
Outliers (%)	0.0	0.05	0.16	0.05
Allowed (%)	2.02	1.74	4.14	2.25
Favored (%)	97.98	98.21	95.7	97.7
B factors (Å^2^)				
Mean	90.9	75.1	76.1	105.3
SD	30.4	30.0	25.2	36.4

### cryoEM Structures of RAD51-ssDNA Filaments in the Presence of Mg^2+^-ATP and Upon ATP Hydrolysis.

**Fig. 3.**
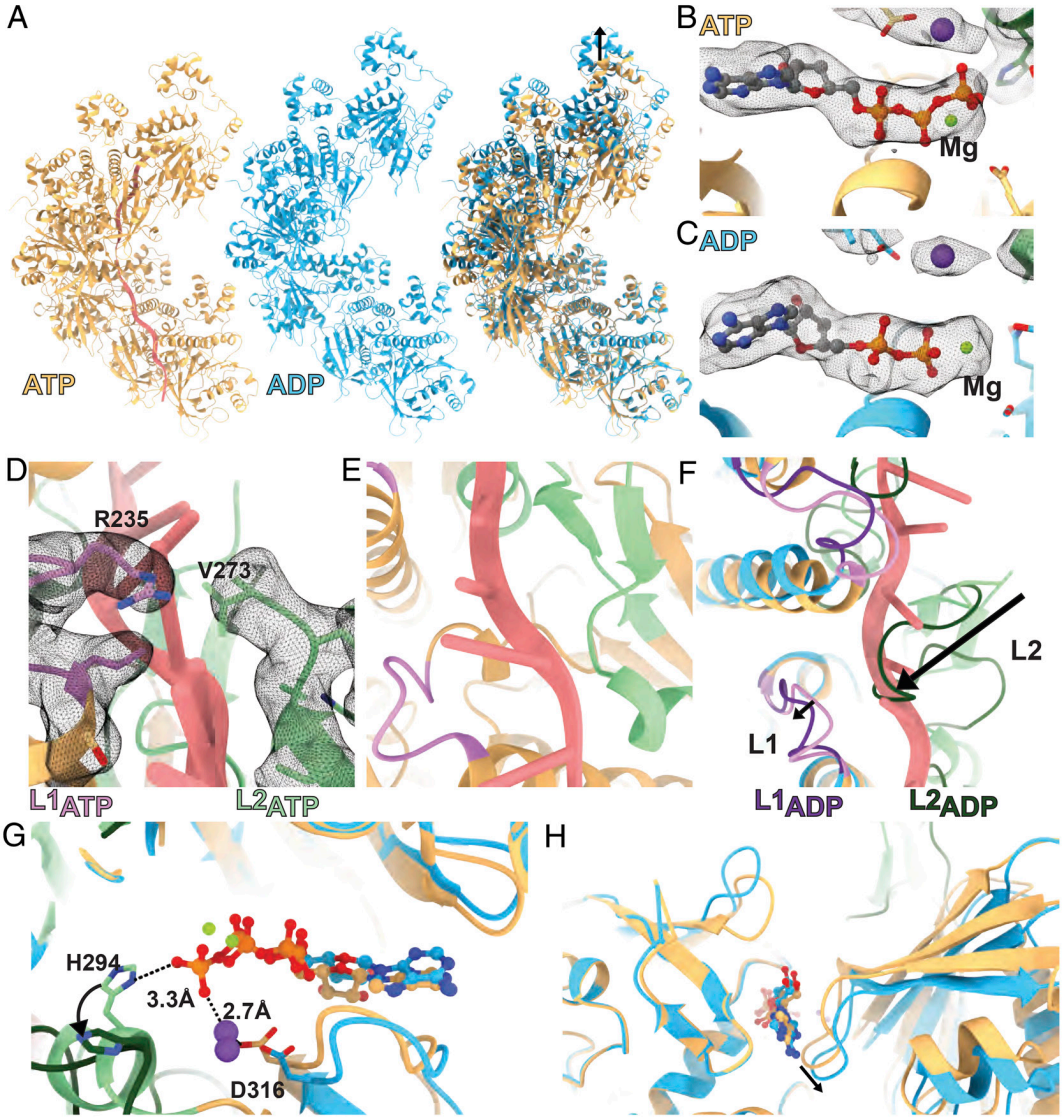
CryoEM structures of Mg^2+^-ATP and Mg^2+^-ADP bound RAD51-ssDNA filaments. (*A*) Comparison of the Mg^2+^-ATP bound RAD51 filament (yellow) with the more relaxed, Mg^2+^-ADP bound filament (blue) and overlay of the two without showing the DNA bound in the Mg^2+^-ATP structure. Arrow indicates the filament stretched in ADP state compared to that of ATP. (*B*) RAD51 nucleotide binding pocket in the ATP-bound state. (*C*) RAD51 nucleotide binding pocket in the ADP-bound state. (*D*) L1 (magenta) and L2 (green) loops of the ATP bound RAD51 filament separate the bound DNA bases (coral) into triplets. (*E*) Interaction of the β-hairpin and the α-helix of the L2 loop (green) with the DNA backbone (coral). (*F*) Overlay of the ATP and ADP bound filaments showing that part of the L2 loop (dark green) in the ADP-bound state is occupying the DNA triplet binding site of the ATP-bound state. (*G*) Overlay of the nucleotide binding sites showing that H294 in the L2 loop (green) coordinates the γ-phosphate of ATP and swings away from the nucleotide when ADP is bound (dark green). (*H*) Conformational changes at the RAD51 dimer interface (arrow) between ATP and ADP states.

Since the ATPase activity of RAD51 plays a key role in HR and RAD51’s ATPase activity is inhibited by Ca^2+^ (37), we optimized conditions including DNA sequences, RAD51 to DNA ratios, and salt concentrations that allow sufficiently stable RAD51-ssDNA filament formation in the presence of Mg^2+^ instead of Ca^2+^. We observed filaments with a range of helical parameters, highlighting the heterogeneity of these filaments. We refined two distinct 3D reconstructions to 3.6 and 3.3 Å resolution (Table 1 and *SI Appendix*, Fig. S3). The two filaments have a rise of 18.5 Å and 16.4 Å, respectively, and a corresponding helical twist of 52.9° and 55.1°, yielding respective helical pitches of 126 Å (6.8 protomers per turn) and 107 Å (6.5 protomers per turn) (Fig. 3*A* and Table 2).

**Table 2.** Filament helical parameters of various RAD51-ssDNA filaments

protein	DNA	Metal ion	Nucleotide	Resolution (Å)	Twist (°)	Rise (Å)	Pitch (Å)	Units per turn	Reference
RAD51	ss	Ca^2+^	ATP	3.8	56.7	16.6	105.4	6.4	(38)
RAD51	ss (no density observed)	Ca^2+^	ADP	3.6	53.1	18.6	126	6.8	(38)
RAD51-BRCA2 (TR2 domain)	ss	Ca^2+^	ATP	2.93	56.2	16.1	103	6.4	(29)
RAD51-RAD51AP1	ss	Ca^2+^	ATP	3.1	55.9	16.0	103	6.4	This study
RAD51	ss	Mg^2+^	ATP	3.3	55.1	16.4	107	6.5	This study
RAD51	ss (no density observed)	Mg^2+^	ADP	3.6	52.9	18.5	126	6.8	This study
RAD51-RAD51AP1 (C29)	ss	Mg^2+^	ATP	3.1	55.8	15.9	103	6.5	This study

Detailed inspections of the two reconstructions revealed that the filament with a shorter pitch has Mg^2+^-ATP bound while the one with a larger pitch has Mg^2+^-ADP bound, suggesting that in our samples, some ATP has been hydrolyzed, resulting in ADP-bound filaments (Fig. 3 *B* and *C*). The ATP-bound structure is similar to those obtained using Mg^2+^-AMP-PNP or Ca^2+^-ATP, validating the use of nonhydrolyzable conditions for structural studies of RAD51-ssDNA filaments (29, 30). The ssDNA is organized into stacked triplets intercalated by the conserved L1 and L2 loops of RAD51, specifically R235 of L1 and V273 of L2 (Fig. 3*D*). Further, the β-hairpin and the α-helix within the L2 region are also involved in DNA binding (Fig. 3*E*). In the ADP-bound filament, we do not observe clear density for ssDNA, consistent with those observed in the Ca^2+^-ADP structures and earlier studies showing that ADP-bound filaments having weaker DNA binding, DNA has dissociated, either fully or partially (31, 38).

Comparing the two structures, we observe the unfolding of the L2 region, losing its α-helix adjacent to the γ-phosphate as well as the β-hairpin, in the ADP bound RAD51 filament (Fig. 3*F*). In the ATP-bound structure, the γ-phosphate helps to coordinate the H294 side chain at the end of the small α-helix of L2 (Fig. 3*G*). Loss of the γ-phosphate leads to the rotation of H294 side chain and subsequent reorganization of the nucleotide-binding pocket. Consequently, the α-helix becomes unfolded and the L2 loop is reconfigured with the β-hairpin becoming a random coil (Fig. 3*F*). The L1 is also positioned slightly away from the ssDNA. These changes in L1 and L2 result in a reduction in DNA binding. In our structure, the L2 tip now occupies the ssDNA position (Fig. 3*F*). However, it is unclear if this repositioning contributes to DNA dissociation or is due to availability of the space vacated by the release of ssDNA. We also observe changes at the protomer interface (Fig. 3*H*), which becomes looser in the ADP-bound form, resulting in the smaller twist angle and higher helical rise between two protomers (Fig. 3 A and *H*).

### cryoEM Structures of the Mg^2+^-ATP Bound RAD51-ssDNA in Complex with the RAD51AP1 C-terminal Domain.

**Fig. 4.**
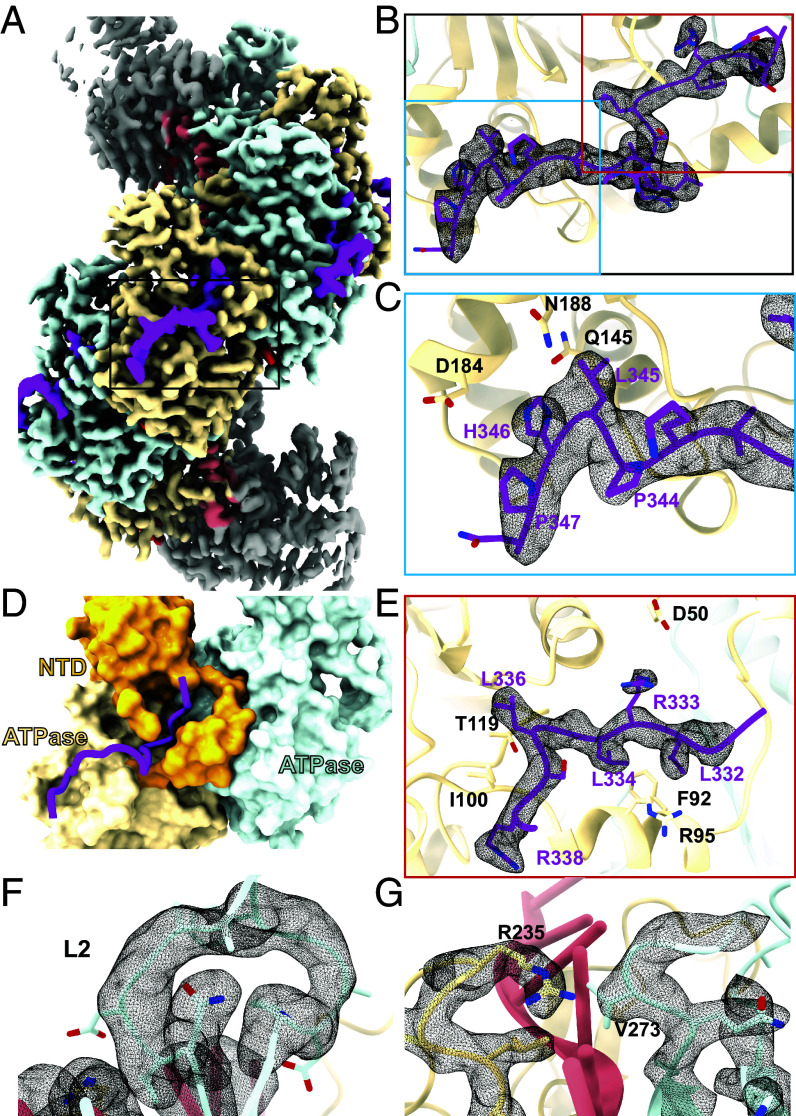
CryoEM structure of the Mg^2+^-ATP bound RAD51 filament in complex with the RAD51AP1 C29 fragment. (*A*) CryoEM map of the RAD51 (yellow and blue) filament on ssDNA (coral) bound by C29 (magenta). (*B*) Closeup view of the density corresponding to RAD51AP1. (*C*) Closeup view of the density corresponding to RAD51AP1 Site-C. (*D*) Surface representation of RAD51 showing RAD51AP1 (magenta) binding in a groove created between the N-terminal domain and the ATPase domain of RAD51. (*E*) Closeup view of the density corresponding to RAD51AP1 Site-M. (*F*) Fully resolved density of the L2 loop of RAD51 when bound to C29. (*G*) Residues of the L1 and L2 loop that separate the bound DNA bases into triplets.

The quality of density corresponding to RAD51AP1 is limited, probably due to the heterogeneity arising from the two RAD51-binding sites across two RAD51 monomers, and Site-N having a weaker affinity for RAD51. Given that mutation of Site-N had limited effect on binding (Figs. 1*B* and 2*D*), we designed a shorter construct that lacks Site-N but contains the major binding site Site-C (C29, C-terminal 29 residues). We reasoned that C29 would bind more uniformly to every RAD51 protomer in the filament, improving occupancy and homogeneity of the samples. The structure was resolved to 3.0 Å resolution (*SI Appendix*, Fig. S4), clearly revealing Mg^2+^-ATP, ssDNA (*SI Appendix*, Fig. S4 *B* and *C*), and density corresponding to RAD51AP1 (Fig. 4 *A* and *B*).

The filaments have a helical twist of 55.8° and a rise of 15.9 Å, giving rise to a helical pitch of 103 Å and 6.5 protomers per turn (Table 2). This is similar to the Ca^2+^-ATP filament bound to full-length RAD51AP1 (104 Å and 6.4 protomers per turn), further supporting the notion that C29 contains the major sites responsible for RAD51 filament modulation. The previously identified H346 in Site-C inserts into a shallow groove on the RAD51 surface, interacting with the side chain of D184 and the main chain of Q145 of RAD51 (Fig. 4*C*), explaining its crucial roles in binding (Fig. 1 *B*–*D*). The adjacent L345 also inserts itself into the same shallow groove. Interestingly, both in the structure of full-length RAD51AP1 in complex with Ca^2+^-ATP-RAD51-ssDNA (Fig. 2*I*) and that of the C29-bound Mg^2+^-ATP-RAD51-ssDNA, we observe continuous density located N-terminal to Site-C (Fig. 4*B*). Indeed, the density resides in a groove created between the RAD51 N-terminal helical bundle and the N-terminal helix (Fig. 4*D*). This site contains the ^332^LRLGLSR^338^ motif, hereafter referred to as Site-M, which inserts itself into a pocket lined by largely hydrophobic residues including RAD51-F92, R95 (surrounding RAD51AP1 L334), I100, and T119 (surrounding RAD51AP1 L336) (Fig. 4 *D* and *E*). Previous yeast two-hybrid analysis demonstrated that L336Q (L319Q in refs. 16 and 23) severely disrupts the interaction between RAD51 and RAD51AP1, and this was confirmed in binding assays with purified proteins (16), consistent with our structure here. Charge interactions between RAD51AP1-R333 and RAD51-D50 accommodate R333 in the pocket and help to stabilize the RAD51 N terminus, now with density visible for residues 19 to 20 above Site-M (*SI Appendix*, Fig. S4*F*); these residues were absent in the structures without RAD51AP1 bound, suggesting that RAD51AP1 binding can also stabilize the RAD51 N terminus.

Looking closer into the structural changes within RAD51, we observe that upon RAD51AP1 binding, the binding pocket, especially around Site-M, remodels to accommodate the peptide. Interestingly, we now observe density for the entire L2 region (Fig. 4*F*), for which the density of the tip was missing in the RAD51-ssDNA structures, suggesting L2 adopts a more rigid conformation upon RAD51AP1 binding. RAD51AP1 binding induces changes such that β-hairpin of the L2 loop is brought closer to DNA, strengthening their interactions, and subsequently stabilizing the tip of the L2, re-enforcing the interactions between V273, the DNA base, and R235 of L1 (*SI Appendix*, Fig. S4*G*).

### RAD51AP1 Modulates RAD51 DNA Binding, Filament Stabilization, and Strand Invasion Via Three Distinct Sites.

**Fig. 5.**
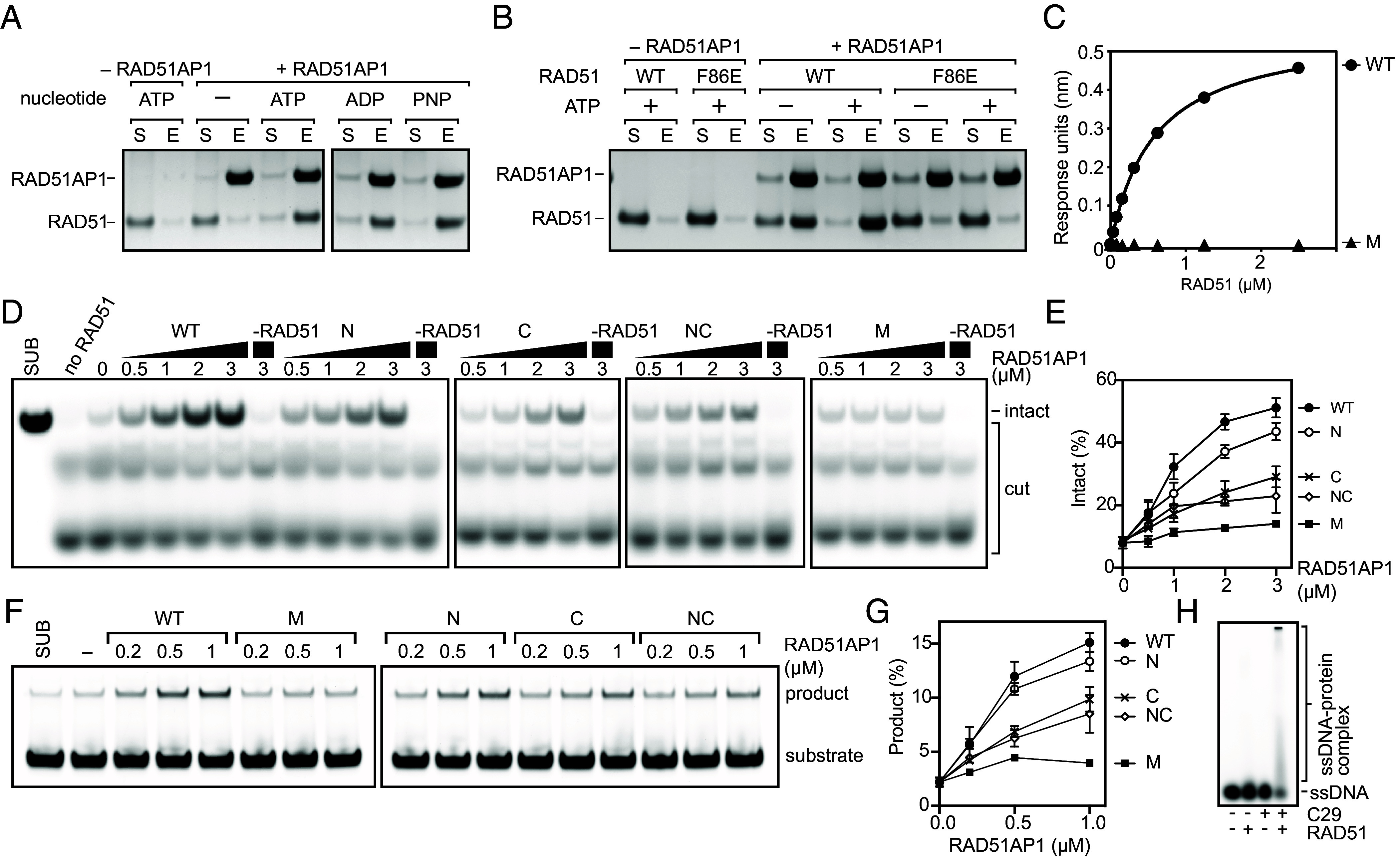
Integrity of Site-M is required for RAD51AP1 activities. (*A*) Pull-down experiments of full-length RAD51AP1 (6xHis-tagged, 1 μM) and RAD51 (1 μM) in the presence of the indicated nucleotides. (*B*) Pull-down experiment of full-length RAD51AP1 (2 μM) with either RAD51 (2 μM) or the oligomerization defective mutant RAD51-F86E (2 μM), with and without ATP. (*C*) Binding curves of RAD51AP1 (wild type and Site-M mutant) obtained from BLI. The wild-type curve shown as a control is the same as in *SI Appendix*, Fig. S1*D*. (*D*) Representative gel images showing RAD51 filament stabilization by full-length RAD51AP1 (wild type or mutants). (*E*) Quantification of (*D*). Averages are shown with error bars depicting SD (n = 3). (*F*) Representative gel image showing the stimulation of RAD51-driven strand exchange by full-length RAD51AP1 (wild type or mutants). (*G*) Quantification of (*F*). Averages are shown with error bars depicting SD (n = 3). (*H*) Representative gel image showing the electrophoretic mobility of RAD51-bound 10 nt ssDNA, with and without RAD51AP1 C29.

To corroborate the structural observations and the functional relevance of the interactions, especially the role of Site-M, we investigated the effects of RAD51AP1 binding on RAD51 HR functions using mutagenesis and biochemical methods. Given that nucleotides bind in-between two RAD51 protomers, we tested whether the nucleotide influences RAD51AP1 binding to RAD51. The omission of ATP and Mg^2+^ in the pull-down buffer led to a complete loss of RAD51 binding (Fig. 5*A* and *SI Appendix*, Fig. S5*A*), supporting the model that RAD51AP1 binds across two RAD51 protomers. This is further supported by the lack of binding to the RAD51-F86E mutant (Fig. 5*B*), which is severely defective for oligomerization (39).

Site-M binds at the dimer interface and its binding induces conformational changes and remodels the filament, suggesting a crucial role in both binding and remodeling. We mutated key residues in Site-M (RLGL to AAGA) and characterized its effects on RAD51 binding, nuclease protection, and strand invasion. Through BLI experiments, we found the Site-M mutant to be severely defective in RAD51 binding, even more so than the Site-C mutant (Fig. 5*C* and *SI Appendix*, Fig. S1*D*). In the nuclease protection assay, the Site-N mutation, which had no obvious effect on RAD51 binding (Figs. 1 *B*–*D* and 2*D*), only caused a minor reduction in stabilization compared to the wild-type protein. By contrast, mutation of Site-C reduced binding, and as expected for a direct binding defect, the impairment in filament stabilization could be overcome by increasing the concentration of RAD51AP1 (Fig. 5 *D* and *E*). The mutation of both sites together further reduced the maximum stabilization that could be achieved (Fig. 5 *D* and *E*). Consistent with the associated binding defect, mutation of Site-M rendered nuclease protection by RAD51AP1 ineffectual (Fig. 5 *D* and *E*). Moreover, the Site-M mutant was severely impaired in the stimulation of RAD51-driven DNA strand exchange (Fig. 5 *F* and *G*). The Site-C mutation resulted in a moderate reduction of strand exchange. As in the nuclease protection assay, this could be compensated for by increasing the concentration of RAD51AP1 (Fig. 5 *F* and *G*).

We observed similar effects in the C59 construct, which lost binding to RAD51 when Site-M was mutated (*SI Appendix*, Fig. S5 *B* and *C*). Wild-type C59 was able to achieve maximal RAD51 filament stabilization in the nuclease protection assay, albeit at higher concentrations compared to full-length RAD51AP1 (*SI Appendix*, Fig. S5 *E* and *F*). Similarly to the full-length protein, the Site-M mutant in the C59 context was defective for nuclease protection (*SI Appendix*, Fig. S5 *E* and *F*). These results agree with our structural observations indicating that the C-terminal region of RAD51AP1, and in particularly Site-M, constitutes the critical module responsible for RAD51 regulation.

To investigate whether RAD51AP1 could indeed promote RAD51 association with DNA as suggested by the structure, especially due to the changes in the L2 region (Fig. 4*F*), we measured RAD51 binding to a short ssDNA (10 nt) (Fig. 5*H*). Since each RAD51 can bind to three nucleotides, 10 nt could accommodate a maximum of 3 RAD51 molecules; we reasoned that this would prevent stable filament formation and therefore allow for the measurement of DNA binding *per se,* rather than binding and subsequent filament formation. As expected, the binding is weak with significant unbound DNA remaining. However, the amount of shifted DNA increased in the presence of C29, suggesting that C29 enhances the initial association of RAD51 with ssDNA.

## Discussion

### ATP Hydrolysis Relaxes Rather Than Compresses RAD51 Filaments.

Thus far, most high-resolution RAD51 filament structures were determined without hydrolyzable ATP, either Ca^2+^-ATP or Mg^2+^-AMPPNP, and all showed similar structures with a helical pitch around 105 Å and 6.4 protomers per turn (Tables 1 and 2). Our Mg^2+^-ATP bound RAD51-ssDNA filament displayed a slightly more relaxed filament (107 Å and 6.5 protomers per turn). The ATP-bound filament has been shown to form on presynaptic (ss) and postsynaptic (ds) DNA in a similar fashion, with DNA stretched between B-form nucleotide triplets, the ATP bound RAD51-ssDNA is therefore ready for pairing with complementary strand. Single molecule experiments showed that dsDNA is stretched upon addition of RAD51 and ATP, consistent with RAD51–ATP binding stretching the DNA (40). Recently published structures of the Ca^2+^-ADP bound (38) and Mg^2+^-ADP bound filaments (41) showed a more extended filament conformation, although the Mg^2+^-ADP structure had two filaments intertwined (41). In that structure, each single filament is extended to a helical pitch of ~145 Å (38, 41). Our Mg^2+^-ADP bound structure has a helical pitch of 126 Å, almost identical to that of Ca^2+^-ADP filaments. These structural data therefore reveal that ATP hydrolysis relaxes RAD51 filaments, contrary to previous single molecule experiments that suggested that ATP hydrolysis contracts the filaments (26). However, that single molecule study was based on DNA length measurements. Indeed, another single molecule study monitoring both RAD51 binding and DNA length showed that ADP-bound RAD51 can bind to dsDNA but does not extend it (40); in comparison to ATP-bound RAD51 filaments where DNA is stretched, the DNA appeared to be more compact in the ADP-bound filaments. Intriguingly, DNA stretching is shown to slow down RAD51 dissociation posthydrolysis (31). These seemingly conflicting results can be reconciled by the conformational changes observed in our ADP-bound filament structure. RAD51–ATP binding stretches dsDNA, enabling stable interactions between L1/L2 loops and DNA. ATP hydrolysis now further stretches the RAD51 filaments, breaking the interactions with DNA, enabling the stretched DNA to return to the preferred B-form, resulting in DNA compaction observed in ref. 40. When the DNA is stretched, the L1/L2 loops in the extended ADP filaments could intercalate between stretched bases, therefore reducing the dissociation from DNA posthydrolysis (31).

Interestingly, the Ca^2+^-ATP bound ssDNA filaments, either alone or in complex with RAD51AP1 or TR2 (the C-terminal domain of BRCA2) (29), have an even smaller helical pitch (103 to 104 Å) and higher twist (6.4 protomers per turn), suggesting an even more tightly packed filament compared to Mg^2+^-ATP (107 Å and 6.5 protomers per turn) (Table 2), Very recently, a crystal structure using a RAD51 dimer construct, which lacks N-terminal domains and with a TR2 peptide fused to one RAD51 protomer, showed a significant rearrangement of the RAD51 dimer compared to those observed in filaments (42). It was proposed that TR2 acts as a clamp for a RAD51 dimer to change its DNA binding specificity from ssDNA to dsDNA (42), therefore promoting its recruitment to stalled replication fork. It appears that TR2 could bind to both RAD51 dimers in solution and in a RAD51 filament, via different interactions although the density for part of the TR2 in the TR2–RAD51 filament structure is poor (29, 42). Nevertheless, these data suggest that many pro recombinases bind at or across RAD51 dimers and stabilize the RAD51 filaments by tightening the filaments, promoting RAD51 recombination activities. Whereas ATP hydrolysis relaxes the filaments, allowing DNA to resume the stable B-form. This could be important for processes postsynapsis, especially those involving modulators such as RAD54 and RTEL but also RAD51AP1 (17), which bind to B-form dsDNA (43).

### Structural Basis of RAD51–DNA Association in the Presence of ATP.

RAD51-filaments are stabilized by DNA interactions via the L1 and L2 loops, the protomer–protomer interactions mediated by the ATPase domains, and the N-terminal domain, which bridges the two ATPase domains (Fig. 4*D*). In the Mg^2+^-ATP and Mg^2+^-ADP bound RAD51 filament structures presented here (Fig. 3), we observe major conformational changes in the L2 regions, similarly to those observed in the Ca^2+^-ATP or Ca^2+^-ADP filaments (38). Interestingly, in that study, the γ-phosphate is shown to coordinate a second metal ion which is absent in the ADP-bound form, and it was thus proposed that this second ion contributed to the folding of L2 (38). In our studies, we observed density corresponding to the second ion in both structures (Fig. 3 *B*-*C*). The second ion interacts with both γ-phosphate and D316, which does not change in the ADP-bound structure. The density corresponding to the second ion in the ADP structure is weaker, which could result from reduced affinity due to the loss of γ-phosphate, or because the resolution of the reconstruction is lower. It is possible that the differences observed are due to the presence of Ca^2+^ or Mg^2+^. However, H294 in the short α-helix of L2 does not interact with the second metal ion in the ATP-bound state, and its reorientation seems to be entirely due to the loss of γ-phosphate, suggesting that the second ion might not affect the folding state of this α-helix (Fig. 3*G*). An earlier crystal structure of RadA also observed a second ion at this position, where it was proposed to play a role in coordinating and polarizing the catalytic water (44). It is possible that the second ion plays a similar catalytic (as opposed to structural) role in RAD51. Indeed, K+ or Na+ both can stimulate ATPase activity (*SI Appendix*, Fig. S6*A*).

### RAD51AP1 Binds to RAD51 Using a Unique Binding Mode.

**Fig. 6.**
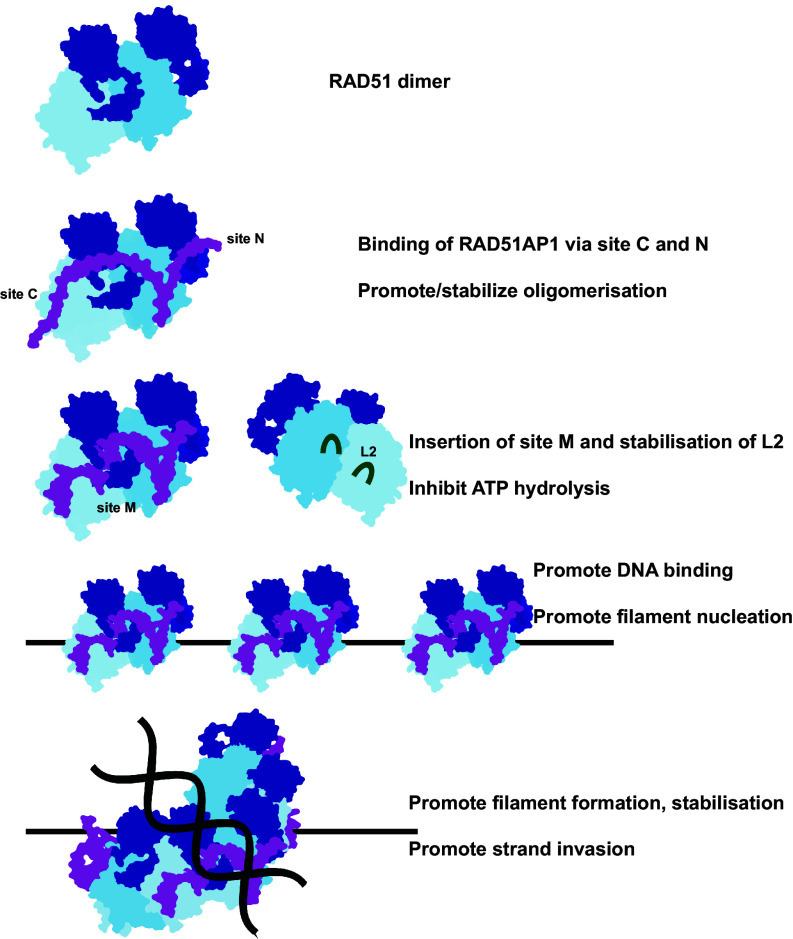
A proposed model for RAD51AP1 in modulating RAD51 activities. Cartoon model of RAD51AP1 binding to the RAD51 dimer, using Site-N and Site-C, promoting oligomerization. Site-M then inserts and strengthens the interface and promotes the ordering of the L2 loop, stabilizing DNA binding and filament nucleation. Once the filament is assembled, the RAD51AP1 bound filament will be more stable and efficient in strand exchange.

Our data here reveal that RAD51AP1 binds across two RAD51 protomers in the RAD51-ssDNA filament via three distinct sites. Site-C, which binds at the RAD51 surface, acts as a recruitment anchor to bring RAD51AP1 onto the RAD51 filaments, allowing Site-N to bind to an adjacent protomer and Site-M insertion (Fig. 6). Site-M is the most important site as the mutant does not bind to RAD51 and is unable to promote filament stabilization or stimulate strand exchange. We propose that Site-N mainly plays a role in filament nucleation via its crucial role in facilitating the binding across two protomers (Fig. 6). However, we postulate that Site-N is less important for other aspects of filament stabilization, and this is supported by our biochemical assays, where its defects were milder than the Site-C/M mutants, and the structural similarities of the C29-bound RAD51 filament, which completely lacks Site-N, and the full-length RAD51AP1-bound filament. Since Site-M inserts itself into a deep pocket on the RAD51 filament surface that is not readily accessible, we propose that Site-C, which binds at the RAD51 surface, acts as a recruitment anchor, which then allows Site-M to be in close proximity to the deep pocket. The subsequent insertion into the RAD51 N-terminal domain then leads to remodeling of the protomer–protomer interface (Fig. 6). Mutating Site-C severely impairs binding, but its defects are rescued at sufficiently high RAD51AP1 concentrations, consistent with its recruitment function. By contrast, mutating Site-M completely abolished all functions, supporting the notion that it is the major binding and remodeling site.

We propose that the Site-M-dependent remodeling of RAD51 in solution promotes its association to ssDNA (Fig. 6). Notably, RAD51AP1 binding induces the stabilization of the L2 loop, promoting DNA binding (Fig. 5*H*). In RecA, I197 in L2 intercalates into the nucleotide triplets in the invading ssDNA while F203 inserts into donor dsDNA, with the aromatic side chain of F203 facilitating stacking interactions with DNA bases and maintaining the D-loop structure, as shown in the RecA strand-exchange intermediate structures (45). This region is highly conserved in RAD51, with L2 loops adopting similar conformations in our C29-bound RAD51-ssDNA filaments (*SI Appendix*, Fig. S6*B*). RAD51 V293 can be superimposed with I197 of RecA while F297 in RAD51 is equivalent to RecA’s F203, suggesting similar roles. Indeed the recently determined RAD51 D-loop structure confirms this (*SI Appendix*, Fig. S6*B*) (46). In the C29-bound structure, the stabilized L2 loop would thus also stimulate strand invasion and D-loop formation. RAD51AP1 therefore stimulates strand exchange by both stabilizing filaments and optimizing L2 conformation for strand invasion and D-loop stabilization (Fig. 6).

Site-M inserts into a pocket formed by the RAD51 N-terminal domain, thus stabilizing the N-terminal domain and protomer–protomer interface. Furthermore, ATP/ADP in the filament is located in-between the RAD51 protomers, with the phosphates buried deep inside the filament. There is a narrow channel where the γ-phosphate could be released upon hydrolysis (*SI Appendix*, Fig. S6*C*). However, this channel is now blocked by Site-M and tightened by the effect of RAD51AP1 binding on the helical parameters of the filament, suggesting that RAD51AP1 binding might also hinder γ-phosphate release, which would inhibit ATP hydrolysis and further stabilize the filament. Consistent with this, we observed reduced ATPase activity in the presence of RAD51AP1 (*SI Appendix*, Fig. S6*D*). Furthermore, in the samples of RAD51-ssDNA-Mg^2+^-ATP in complex with C29, we do not observe filaments with larger helical pitches that correspond to ADP-bound filaments, consistent with inhibition of γ-phosphate release. Previous biochemical analysis with mouse proteins also showed that RAD51AP1 can reduce ATP hydrolysis by RAD51 (47). This effect may further stabilize RAD51 and promote filament formation as well as stabilize subsequently formed filaments (Fig. 6).

Comparison with other RAD51 interacting partners reveals the differences and similarities in their binding modes. Site-N is predicted to bind to RAD51 via a WVPP motif, which is reminiscent of the FQPP motif employed by the C-terminal region of BRCA2 (TR2) to bind to RAD51 (*SI Appendix*, Fig. S6*E*) (29, 42). The TR2–RAD51 complex has been structurally characterized in both filament (29) and nonfilament (42) states using cryoEM and X-ray crystallography respectively. In both complexes, FQPP interact with RAD51 surfaces and in the crystal structure, additional residues adjacent to FQPP stabilize the two monomers (42). RAD51AP1 Site-C also shares a binding site with BRCA2 TR2, suggesting that both RAD51AP1 Site-N and Site-C would overlap with TR2 binding. Recent studies reveal that FIGNL1 binds at three distinct RAD51 sites. Two sites within the FRBD domain are predicted to overlap with BRCA2 BRC4 and TR2 binding sites, respectively. Uniquely, FIGNL1 encloses the RAD51 N terminus in its hexamer pore formed by its C-terminal ATPase domains (14). Site-M does not overlap with the binding of TR2, FIGNL1, or BRC4. It will be interesting to investigate whether these modulators compete for binding to RAD51, or act synergistically, and at what stoichiometry multiple modulators decorate RAD51 filaments during HR.

### RAD51AP1 Is a Versatile RAD51 Modulator and Remodels RAD51 Filaments.

Our comprehensive data here reveal the molecular mechanism of RAD51AP1 in promoting RAD51 activities in HR. We showed that via three binding sites across two RAD51 monomers, RAD51AP1 modulates almost all RAD51 activities in HR: enhancing DNA binding, filament nucleation, filament stabilization, and strand exchange. In addition to modulating RAD51 filaments, RAD51AP1 has been proposed to act as a bridge in tethering RAD51-ssDNA filaments formed at the DSB site to the sister chromatid. Its ability to bind to TERRA and promote R-loop formation has highlighted its roles in ALT and telomere maintenance. Whether these activities are independent or act synergistically with its RAD51 modulator function, which activities are important for the different processes RAD51AP1 is involved in, and how its versatile activities are regulated, remain to be investigated.

## Materials and Methods

### Sequence Alignment.

A multiple sequence alignment of RAD51AP1 sequences of representative animals was generated using clustal omega (48) and a sequence logo was generated using the WebLogo server (49).

### Cloning.

The RAD51AP1 sequence was ordered as a synthetic gene from Invitrogen, amplified by PCR using the Cloneamp mastermix (Takara) and inserted into a pET28 vector with an N-terminal sumo tag and a C-terminal His tag. Substitution mutations and truncations were generated by PCR and the fragments were combined using the NEBuilder HiFi assembly kit (NEB).

The RAD51 F86E plasmid was generated from a pCH1 RAD51 plasmid (50). The plasmid for coexpression of BRC4 with RAD51 (pJ9) was generated by amplifying 6xHis-MBP-BRC4 (14) by PCR and inserting it in the pCH1 plasmid upstream of RAD51.

### Protein Purification.

RAD51 proteins were expressed in BL21 (DE3) cells carrying the plasmid grown in 500 mL of LB media supplemented with kanamycin (50 μg/mL) at 37 °C to an optical density of 0.4 and then transferred to 18 °C. Protein expression was induced 1 h later by addition of 0.5 mM IPTG. Cells were harvested by centrifugation and stored at −20 °C.

RAD51 was purified using an MBP-BRC4 construct as described previously with some modifications (51). Briefly, RAD51, His-MBP-BRC4, and GroEL were coexpressed from a single plasmid. After initial pulldown using a HisTrap column (Cytiva^TM^), RAD51 was further purified with a HiTrap Heparin column (Cytiva^TM^) and a HiTrap Q anion exchange column (Cytiva^TM^). Peak fractions were concentrated and flash frozen in liquid nitrogen.

RAD51 F86E was purified as described previously (52) except that the final purification was carried out using size-exclusion chromatography with an S200 increase column (Cytiva^TM^) in 50 mM Tris pH 7.5, 150 mM KCl, 1 mM EDTA, 0.5 mM TCEP.

Full-length RAD51AP1 and mutants were expressed from a pET28 vector including an N-terminal SUMO tag and a C-terminal His tag. Cells were lysed by sonication and lysates were clarified by centrifugation. Purifications were achieved via HisTrap column, followed by HiTrap Heparin column, HiTrap Q column before gel filtration. Detailed protocols can be found in *SI Appendix*, *Supplementary Methods*.

### Pull-Down Assays.

30 µL His-tagged RAD51AP1 (1 µM of full-length or 2 µM of C59) was immobilized on magnetic cobalt resin by incubating at 37 °C for 10 min with mixing. A magnetic stand was used to discard the supernatant. The protein-bound resin was then resuspended in 30 µL of pull-down buffer containing RAD51 (1 µM for full-length and 2 µM for C59) and incubated with mixing at 37 °C for 5 min. The supernatant containing unbound RAD51 was recovered and bound proteins were eluted by incubation with SDS-PAGE loading buffer (65 °C, 1,300 rpm, 5 min). The protein content of both fractions was analyzed by SDS-PAGE, and gels were imaged using a BioRad imager. FIJI was used for quantification (53). Briefly, the images were background subtracted using the rolling ball method (50 pixels), then the intensity of RAD51 and RAD51AP1 bands in the supernatant and eluate was determined. The percentage of signal in the eluate was calculated. For each experiment, the value for the nonspecific RAD51 binding (minus RAD51AP1 sample) was subtracted from all samples, then the amount of RAD51 was normalized to RAD51AP1 binding and expressed relative to wild-type protein. Detailed buffers and protocols are described in *SI Appendix*, *Supplementary Methods*.

### BLI.

BLI experiments were performed on an Octet Red instrument (Sartorius) operating at 25 °C. RAD51AP1 (full-length or C59, wild-type or mutant variants) with a C-terminal hexahistidine tag was diluted to 1 µg/mL with BLI buffer and transferred into a row on a 96-well plate (200 µL per well, x8). RAD51 was serially diluted (twofold) with BLI buffer from 2.5 µM to 39 nM, and along with a buffer alone sample, 200 µL of each concentration was transferred into a separate row. Nickel-NTA biosensors (Sartorius cat. # 18-5101) were soaked in BLI buffer for 20 min prior to experiments. Subsequent steps are automated. See *SI Appendix*, *Supplementary Methods* for details. Data were analyzed using Octet BLI Analysis software (Sartorius) and in-house software (54). Equilibrium dissociation constants (K_d_) were estimated from the instrument response against RAD51 concentration using least squares nonlinear regression and a 1:1 binding model. Experiments were repeated in triplicate and errors are reported as SD from the mean.

### Nuclease Protection Assay.

10 µMnt of a Cy5-labeled oligonucleotide (60-mer) was supplemented with RAD51 (2 µM) and the indicated concentration of RAD51AP1. Following a 5 min incubation at 37 °C, 2 µL of DNase I (NEB^TM^ M0303) was added and incubation continued for 15 min. Reactions were then deproteinized by addition of stop solution (final concentration 12 mM Tris-Cl [7.5], 7.52 mM EDTA, 0.45% SDS, 0.75 mg/mL proteinase K). DNA was resolved on 10% PAGE gels and imaged using a Bio-Rad imager (ChemiDoc MP Imaging System^TM^). FIJI was used for quantification (53). Briefly, the images were background subtracted using the rolling ball method (50 pixels). Next, the total lane signal was determined and the fraction of signal corresponding to intact DNA was then expressed as a percentage of the total.

### Negative Stain.

Filaments were formed on oligo BA494 (1.5 μM nucleotides) with RAD51 (375 nM) and C59 (1.5 μM) in buffer D (20 mM HEPES, pH 7.5, 100 mM KCl, 0.5 mM TCEP) supplemented with 2 mM ATP and 5 mM MgCl_2_ at 37 °C for 5 min. 10 μL samples were applied to glow discharged 300 mesh carbon coated copper grids (Agar Scientific), blotted, and stained with 2% uranyl acetate. Micrographs were acquired on a T12 microscope (FEI) at a magnification of 21,000 using a Rio CMOS camera (Gatan^TM^).

Filament lengths were measured as described previously (55).

### Strand Exchange Assay.

RAD51 (1 µM) was incubated with 3 µMnt of a 116-mer oligonucleotide in strand exchange buffer at 37 °C. After 8 min, the indicated concentration of RAD51AP1 were added and the reaction was incubated for another 8 min. RPA (0.2 µM) was then added to the reaction, and following another incubation (37 °C, 8 min), the reaction was initiated via the addition of 1 µMbp 60-mer dsDNA and incubated at 37 °C for 20 min. Reactions were deproteinized, resolved by PAGE, and imaged as above. FIJI was used for quantification (53). Briefly, the images were background subtracted using the rolling ball method (50 pixels). Next, the total lane signal was determined and the fraction of signal corresponding to product was then expressed as a percentage of the total. This was then expressed relative to the RAD51 alone reaction to yield fold stimulation.

### ATPase Assay.

3 µM RAD51, 9 µMnt poly-dT ssDNA (72-mer) and 1 µM RAD51AP1 where indicated were incubated in buffer at 37 °C for 90 min. The assay including RAD51AP1 was run in the presence of 50 mM KOAc at 37 °C for 70 min. Reactions were stopped by addition of 20 mM EDTA then diluted four-fold to reduce phosphate concentration. A commercial colorimetric kit was then used following the manufacturer’s instructions (MAK307, Merck) to determine phosphate concentration.

### Electrophoretic Mobility Assay.

1 µM LK_M10 in buffer D containing AMP-PNP (2 mM) and MgCl_2_ (5 mM) was mixed with RAD51 (4 µM) and C29 (10 µM) or buffer as indicated. After incubation for 5 min at room temperature the mixture was resolved by native PAGE.

### cryoEM Sample Preparation.

#### RAD51AP1 bound to RAD51 filament formed with Ca^2+^-ATP.

Filaments were formed on oligo LK_M50 (12 μM nucleotides) with RAD51 (4 μM) in buffer D supplemented with 2 mM ATP and 5 mM CaCl_2_ at 37 °C for 5 min. Immediately before preparing grids, the filaments were diluted to a final concentration of 1.6 μM RAD51. 2 μL of filaments were applied to glow discharged 300 mesh carbon coated lacey carbon grids (LC300-Au-UL, EM Resolutions) in a Mark IV vitrobot (ThermoFisher Scientific^TM^) and incubated at 24 °C, 100% humidity for 3 min. 1 μL of sample was removed from the grid and 2 μL of RAD51AP1 (0.8 μM in buffer D supplemented with 2 mM ATP and 5 mM CaCl_2_) was applied to the grid and incubated for 1 min. Finally, 2 μL were removed and replaced with 3 μL of buffer and incubated for 1 min. Grids were then blotted and plunged into liquid ethane.

#### RAD51 filament formed with Mg^2+^-ATP.

Filaments were formed on oligo LK_AAC60 (21 μM nucleotides) with RAD51 (10.5 μM) in buffer D supplemented with 3 mM ATP and 7.5 mM MgCl_2_ at 37 °C for 5 min. They were then diluted 2:1 with buffer D containing ATP and MgCl_2_ to a final RAD51 concentration of 7 μM. 4 μL of filaments were applied to R2/2, 200 mesh Ultrafoil grids (EMS^TM^), which had been washed in chloroform before use, in a Mark IV vitrobot (ThermoFisher Scientific^TM^) at 4 °C, 100% humidity, and blotted and plunged into liquid ethane after 30 s.

#### C29 bound to RAD51 filament formed with Mg^2+^-ATP.

The filaments were formed as above and then diluted 2:1 with C29 in buffer D containing ATP and MgCl_2_ to final RAD51 concentration of 7 μM and a final concentration of C29 of 28 μM. The grids were then prepared in the same manner as for the apo filament.

All grids were imaged at the LonCEM facility at the Francis Crick Institute on a Titan Krios microscope equipped with a K3 detector (Gatan^TM^) at a pixel size of 1.08 Å. Full acquisition details are in Table 1.

### cryoEM Data Processing.

For all datasets, movies were processed with MotionCor2 as implemented in relion5 (56). All subsequent processing was done in cryoSPARC version 3-4.6 (57). Detailed processing protocol can be found in *SI Appendix*, *Supplementary Methods*.

### Model Building.

Models of 6 RAD51 protomers with ssDNA, ATP or ADP, Ca^2+^ or Mg^2+^ and RAD51AP1 C29 as appropriate were modeled in AlphaFold (36) and docked into the maps as rigid bodies. The models were partially manually rebuilt in Coot (58). The final models were refined using phenix.real_space_refine (59).

### Estimation of Helical Parameters.

Each map was split to extract the central part in which the model had been built using ChimeraX (60). This map was then imported into cryoSPARC and the helical parameters were estimated using the job symmetry search utility.

## Supplementary Material

Appendix 01 (PDF)

## Data Availability

Structural models and cryoEM reconstructions’ data have been deposited in PDB [9QN8 (61), 9QNA (62), 9QNB (63), and 9QNC (64)] and EMDB [EMD-53239 (65), EMD-53241 (66), EMD-53242 (67), and EMD-53243 (68)].
