# Supplementary material for: RAD51AP1 is a versatile RAD51 modulator
Source: Proc Natl Acad Sci U S A. 2025 Dec 3;122(49):e2514728122. doi: 10.1073/pnas.2514728122 (PMC12704761; doi:10.1073/pnas.2514728122)
Supplement: Supplementary file 1 — Appendix 01 (PDF) [file pnas.2514728122.sapp.pdf]

PNAS

Supporting Information for

RAD51AP1 is a versatile RAD51 modulator

Lucas Kuhlen[1,2,4], Bilge Argunhan[1,2,4], Pengtao Liang[1,2], Janet Zhong[1], Laura Masino[3], Xiaodong Zhang[1,2*]

[1]Section of Structural and Synthetic Biology, Faculty of Medicine, Imperial College London, London SW7 2AZ, UK

[2]DNA Processing Machines Laboratory, Francis Crick Institute, London NW1 1AT, UK

[3]Structural Biology Science Technology Platform, Francis Crick Institute, UK

[4]These authors contributed equally to this work

[*]correspondence should be addressed to: Xiaodong Zhang

Email: Xiaodong.zhang@imperial.ac.uk

This PDF file includes:

Supplementary methods
Figures S1 to S6
SI References

Supplementary Methods

Protein purification

All proteins were expressed in BL21 (DE3) cells carrying the plasmid grown in 500 ml of LB media supplemented with kanamycin (50 µg/ml) at 37 °C to an optical density of 0.4 and then transferred to 18 °C. Protein expression was induced one hour later by addition of 0.5 mM IPTG. Cells were harvested by centrifugation and stored at -20 °C.

RAD51 was purified using an MBP-BRC4 construct as described previously with some modifications (1). Briefly, RAD51, His-MBP-BRC4 and GroEL were co-expressed from a single plasmid. After initial pulldown using a HisTrap column (Cytiva^{TM}), RAD51 was further purified with a HiTrap Heparin column (Cytiva^{TM}) and a HiTrap Q anion exchange column (Cytiva^{TM}). Peak fractions were concentrated and flash frozen in liquid nitrogen.

RAD51 F86E was purified as described previously (2) except that the final purification was carried out using size-exclusion chromatography with an S200 increase column (Cytiva^{TM}) in 50 mM Tris pH 7.5, 150 mM KCl, 1 mM EDTA, 0.5 mM TCEP.

Full-length RAD51AP1 and mutants were expressed from a pET28 vector including an N-terminal sumo tag and a C-terminal his tag. Cell pellets were resuspended in His binding buffer (20 mM HEPES, pH 7.5, 500 mM KCl, 12.5 mM imidazole, 0.5 mM TCEP) supplemented with denarase nuclease (c-LEcta), salt active nuclease (SERVA) and cOmplete protease inhibitors (Roche^{TM}). Cells were lysed by sonication and the lysate was clarified by centrifugation (50,000 g, 60 minutes) and then applied to a 5 ml HisTrap HP column (Cytiva^{TM}). The column was washed with 20 ml His binding buffer, 10 ml wash buffer 1 (20 mM HEPES, pH 7.5, 1M KCl, 25 mM imidazole, 0.02% DDM, 0.5 mM TCEP), 20 ml wash buffer 2 (20 mM HEPES, pH 7.5, 50 mM KCl, 25 mM imidazole, 0.5 mM TCEP), 10 ml wash buffer 3 (20 mM HEPES, pH 7.5, 50 mM KCl, 300 mM imidazole, 0.5 mM TCEP) and eluted in 10 ml elution buffer (20 mM HEPES, pH 7.5, 50 mM KCl, 500 mM imidazole, 0.5 mM TCEP). The sumo tag was cleaved by adding 100 µg 3C protease and incubating at 4 °C overnight. The protein was further

purified using a 1ml HiTrap Heparin column run in 20 mM HEPES, pH 7.5, 0.5 mM TCEP and KCl, eluting at a concentration of approximately 150 mM KCl. The peak fractions were pooled, diluted in the heparin running buffer and applied to a 1ml HiTrap Q column. The protein eluted at a concentration of approximately 200 mM KCl. Finally, the protein was polished by gel filtration in a superose 6 increase column (Cytiva™) in 20 mM HEPES, pH 7.5, 0.5 mM TCEP and 150 mM KoAc. The protein was concentrated and frozen in aliquots in liquid nitrogen.

RAD51AP1 C59 was expressed from a pET28 vector including an N-terminal sumo tag and a C-terminal his tag. Cell pellets were resuspended in buffer A (20 mM Tris, pH 8, 50 mM NaCl, 0.5 mM TCEP) supplemented with 10 mM imidazole, denarase nuclease (c-LEcta) and cOmplete protease inhibitors (Roche™). Cells were lysed by sonication and the lysate was clarified by centrifugation (75,000 g, 30 minutes) and then applied to a 5 ml HisTrap HP column (Cytiva™). The column was washed with 10 ml buffer B (20 mM Tris, pH 8, 1000 mM NaCl, 0.5 mM TCEP) supplemented with 10 mM imidazole and 0.02% DDM, followed by 15 ml of buffer A supplemented with 50 mM imidazole. Proteins were eluted with buffer A containing 400 mM imidazole. The sample was further purified using a HiTrap Q column (Cytiva™) and eluted in a gradient of 100 mM to 600 mM NaCl in buffer A. Finally, the protein was polished by gel filtration in a superose 6 increase column (Cytiva™) in 20 mM HEPES, pH 7.5, 0.5 mM TCEP and 150 mM KoAc. The protein was concentrated and frozen in aliquots in liquid nitrogen.

C29 was expressed from a pET28 vector with an N-terminal twin strep-sumo tag and a C-terminal His tag. Cell pellets were resuspended in buffer TBK300 (20 mM Tris, pH 7.5, 300 mM KCl, 0.5 mM TCEP) supplemented with denarase nuclease (c-LEcta), salt active nuclease (SERVA) and cOmplete protease inhibitors (Roche™). Cells were lysed by sonication and the lysate was clarified by centrifugation (50,000 g, 30 minutes) and then applied to a 5 ml StrepTrap HP column (Cytiva™). The column was washed with 10 ml wash buffer (20 mM Tris, pH 7.5, 1000 mM KCl, 0.5 mM TCEP), 10 ml TBK300 and eluted in 15 ml TBK300 containing 2 mg/ml desthiobiotin. The twin strep and sumo tag were removed using 100 µg

3C protease, which were added to the eluate and incubated overnight at 4 °C. The sample was then diluted to 100 mM KCl and applied to a HiTrap Q column (Cytiva™). The protein was found in the flow-through, which was concentrated and frozen in liquid nitrogen.

Pull-down assays

Hexahistidine-tagged RAD51AP1 (1 μM of full-length or 2 μM of C59) in 30 μL of pull-down buffer (25 mM Tris-OAc [7.5], 150 mM or 100 mM KOAc [full-length or C59, respectively], 1 mM ATP, 5 mM MgOAc, 0.25 mM TCEP, 0.1 mg/mL BSA, 0.1% Tween 20, 2.5% glycerol, 50 mM imidazole) was immobilized on magnetic cobalt resin (ThermoFisher Scientific™ 10103D) by incubating at 37°C for 10 min with mixing. A magnetic stand was used to discard the supernatant. The protein-bound resin was then resuspended in 30 μL of pull-down buffer containing RAD51 (1 μM for full-length and 2 μM for C59) and incubated with mixing at 37°C for 5 min. The supernatant containing unbound RAD51 was recovered and bound proteins were eluted by incubation with SDS-PAGE loading buffer (65°C, 1300 rpm, 5 min). The protein content of both fractions was analysed by SDS-PAGE and staining (ReadyBlue Sigma-Aldrich), and gels were imaged using a BioRad imager (ChemiDoc MP Imaging System™). FIJI was used for quantification (3). Briefly, the images were background subtracted using the rolling ball method (50 pixels), then the intensity of RAD51 and RAD51AP1 bands in the supernatant and eluate was determined. The percentage of signal in the eluate was calculated. For each experiment, the value for the non-specific RAD51 binding (minus RAD51AP1 sample) was subtracted from all samples, then the amount of RAD51 was normalized to RAD51AP1 binding and expressed relative to wild-type protein.

Bio-layer interferometry (BLI)

Bio-Layer Interferometry (BLI) experiments were performed on an Octet Red instrument (Sartorius) operating at 25 °C. RAD51AP1 (full-length or C59, wild-type or mutant variants) with a C-terminal hexahistidine tag was diluted to 1 μg/mL with BLI buffer (25 mM HEPES [7.5], 150 mM KOAc, 5 mM MgOAc, 1 mM ATP, 0.05% Tween20, 0.5 mM TCEP) and

transferred into a row on a 96 well plate (200 µL per well, x8). RAD51 was serially diluted (two-fold) with BLI buffer from 2.5 µM to 39 nM, and along with a buffer alone sample, 200 µL of each concentration was transferred into a separate row. Nickel-NTA biosensors (Sartorius cat. # 18-5101) were soaked in BLI buffer for 20 min prior to experiments. The following steps were automated. First, the biosensors were dipped in BLI buffer (100 s) for a first Baseline step, then RAD51AP1 was loaded onto the biosensors until a response of 0.1 nm was achieved. Next, the biosensors were transferred into fresh BLI buffer for a second Baseline (40 s), to check RAD51AP1 was stably bound to the sensors. The biosensors were then transferred into the RAD51 dilution series to monitor association and then to fresh BLI buffer to monitor dissociation. Double reference subtraction was performed for all samples by subtracting the curve recorded without RAD51 and the response values recorded with no RAD51AP1 loaded on the sensors. The resultant responses were averaged and then plotted against RAD51 concentration. Data were analysed using Octet BLI Analysis software (Sartorius) and in-house software (4). Equilibrium dissociation constants (K_d) were estimated from the instrument response against RAD51 concentration using least squares non-linear regression and a 1:1 binding model. Experiments were repeated in triplicate and errors are reported as standard deviations from the mean.

Nuclease protection assay

10 µMnt of a Cy5-labeled oligonucleotide (60-mer) in nuclease protection buffer (25 mM Tris-OAc [7.5], 100 mM KOAc, 2 mM ATP, 5 mM MgOAc, 0.5 mM TCEP, 2.5% glycerol) was supplemented with RAD51 (2 µM) and the indicated concentration of RAD51AP1. Following a 5 min incubation at 37°C, 2 µL of DNase I (NEB™ M0303) was added and incubation continued for 15 min. Reactions were then deproteinized by addition of stop solution (final concentration 12 mM Tris-Cl [7.5], 7.52 mM EDTA, 0.45% SDS, 0.75 mg/mL proteinase K). DNA was resolved on 10% PAGE gels and imaged using a Bio-Rad imager (ChemiDoc MP Imaging System™). FIJI was used for quantification (3). Briefly, the images were background subtracted using the rolling ball method (50 pixels). Next, the total lane signal was determined

and the fraction of signal corresponding to intact DNA was then expressed as a percentage of the total.

Strand exchange assay

RAD51 (1 μM) was incubated with 3 μMnt of a 116-mer oligonucleotide in strand exchange buffer (25 mM Tris-OAc [7.5], 100 mM KCl, 2 mM ATP, 1 mM $MgCl_2$, 2 mM $CaCl_2$, 0.5 mM TCEP, 0.1 mg/mL BSA) at 37°C. After 8 minutes, the indicated concentration of RAD51AP1 were added and the reaction was incubated for another 8 minutes. RPA (0.2 μM) was then added to the reaction, and following another incubation (37°C, 8 min), the reaction was initiated via the addition of 1 μMbp 60-mer dsDNA and incubated at 37°C for 20 minutes. Reactions were deproteinized, resolved by PAGE, and imaged as above. FIJI was used for quantification (3). Briefly, the images were background subtracted using the rolling ball method (50 pixels). Next, the total lane signal was determined and the fraction of signal corresponding to product was then expressed as a percentage of the total. This was then expressed relative to the RAD51 alone reaction to yield fold stimulation.

ATPase assay

3 μM RAD51,9 μMnt poly-dT ssDNA (72-mer) and 1 μM RAD51AP1 where indicated were incubated in ATPase buffer (25 mM Tris-OAc [pH 7.5], 0.5 mM ATP, 5 mM MgOAc, 0.5 mM TCEP, 0.01% Tween20, 2.5% glycerol), supplemented with 50 mM salt (KOAc or NaOAc, as indicated), at 37°C for 90 min. The assay including RAD51AP1 was run in the presence of 50 mM KOAc at 37°C for 70 min. Reactions were stopped by addition of 20 mM EDTA then diluted four-fold to reduce phosphate concentration. A commercial colorimetric kit was then used following the manufacturer's instructions (MAK307, Merck) to determine phosphate concentration.

Electrophoretic mobility assay

1 µM LK_M10 in buffer D containing AMP-PNP (2 mM) and MgCl2 (5 mM) was mixed with RAD51 (4 µM) and C29 (10 µM) or buffer as indicated. After incubation for 5 minutes at room temperature the mixture was resolved by native PAGE.

cryoEM sample preparation

RAD51AP1 bound to RAD51 filament formed with Ca^{2+}-ATP

Filaments were formed on oligo LK_M50 (12 µM nucleotides) with RAD51 (4 µM) in buffer D supplemented with 2 mM ATP and 5 mM $CaCl_2$ at 37 °C for 5 minutes. Immediately before preparing grids, the filaments were diluted to a final concentration of 1.6 µM RAD51. 2 µl of filaments were applied to glow discharged 300 mesh carbon coated lacey carbon grids (LC300-Au-UL, EM Resolutions) in a Mark IV vitrobot (ThermoFisher Scientific™) and incubated at 24 °C, 100% humidity for 3 minutes. 1 µl of sample was removed from the grid and 2 µl of RAD51AP1 (0.8 µM in buffer D supplemented with 2 mM ATP and 5 mM $CaCl_2$) was applied to the grid and incubated for 1 min. Finally, 2 µl were removed and replaced with 3 µl of buffer and incubated for 1 minute. Grids were then blotted and plunged into liquid ethane.

RAD51 filament formed with Mg^{2+}-ATP

Filaments were formed on oligo LK_AAC60 (21 µM nucleotides) with RAD51 (10.5 µM) in buffer D supplemented with 3 mM ATP and 7.5 mM $MgCl_2$ at 37 °C for 5 minutes. They were then diluted 2:1 with buffer D containing ATP and $MgCl_2$ to a final RAD51 concentration of 7 µM. 4 µl of filaments were applied to R2/2, 200 mesh Ultrafoil grids (EMS™), which had been washed in chloroform before use, in a Mark IV vitrobot (ThermoFisher Scientific™) at 4 °C, 100% humidity and blotted and plunged into liquid ethane after 30 seconds.

C29 bound to RAD51 filament formed with Mg^{2+}-ATP

The filaments were formed as above and then diluted 2:1 with C29 in buffer D containing ATP and $MgCl_2$ to final RAD51 concentration of 7 µM and a final concentration of C29 of 28 µM. The grids were then prepared in the same manner as for the apo filament.

All grids were imaged at the LonCEM facility at the Francis Crick Institute on a Titan Krios microscope equipped with a K3 detector (Gatan™) at a pixel size of 1.08 Å. Full acquisition details are in **Table 1**.

cryoEM data processing

For all datasets, movies were processed with MotionCor2 as implemented in relion5 (5). All subsequent processing was done in cryoSPARC version 3-4.6 (6).

RAD51AP1 bound to RAD51 filament formed with Ca^{2+}-ATP

5001 micrographs were imported into cryoSPARC. CTF was estimated using PatchCTF and 159 micrographs were rejected based on poor CTF fit resolution worse than 10 Å and defocus lower than 0.3 µm. Particles were picked using template free filament tracer. 2.4 million particles were picked and extracted binned 4 times. After multiple rounds of 2D classification in cryoSPARC, 510,927 particles were selected and re-extracted binned 2 times. After further 2D classification, 489,416 particles were selected and re-extracted without binning. After further 2D classification, 458,792 particles were selected. Homogeneous 3D refinement, without applying helical symmetry, using an initial volume of an AP1 bound filament from a previous screening dataset led to a map of the filament at a resolution of 3.1 Å. A mask was designed around a piece of weak density on the outside of the filament. After three rounds of 3D classification with 10 classes each followed by homogeneous refinement, 137,283 particles were selected and refined with homogeneous refinement, resulting in a map at a resolution of 3.2 Å. Further minimizing per-particle scale and per-particle defocus improved resolution to 3.1 Å. The interpretability of the map was further improved using EMReady (7). Final helical parameters were estimated based on the central part of the map in which the

atomic model was built, giving rise to final estimates of a helical rise of 16 Å and a helical twist of 55.9°.

RAD51 filaments formed with Mg^{2+}-ATP

3096 micrographs were imported into cryoSPARC. CTF was estimated using PatchCTF and 224 micrographs were rejected based on poor CTF fit resolution worse than 10 Å and defocus lower than 0.3 µm. 6.6 million particles were picked using a combination of template free filament tracer and template picker and template based filament tracer using 2D averages of a RAD51 filament from a previous screening dataset as templates. After removing overlapping particles, 5.6 million particles were extracted in a small box (180 pixels) and after 2 rounds of 2D classification 983,859 particles were selected and re-extracted in a larger box (256 pixels), binned 2 times. After further 2D classification, 538,943 particles were selected and refined with helical refinement, using a reconstruction of the RAD51AP1 bound Ca^{2+}-ATP RAD51 filament as an initial volume and initial helical twist estimate of 56° and a rise estimate of 16 Å. Refinement of the helical parameters during helical refinement led to revised estimates of 54.6° and 16.9 Å respectively. These were used as input values into another helical refinement, resulting in final estimates of 54.1° and 17.3 Å in the consensus refinement. The particles were classified in 3D into 5 classes and the particles re-extracted without binning and then subjected to helical refinement again. The 3D classes were grouped into two groups, one with high helical rise and one with low rise and particles belonging to each group were combined and refined again. They were then classified again in 2D and 229,610 particles belonging to the high helical rise conformation and 242,944 particles belonging to the low helical rise conformation were selected. Final helical refinement resulted in volumes at resolutions of 3.6 Å and 3.3 Å respectively. The interpretability of the maps was further improved using EMReady (7). Final helical parameters were estimated based on the central part of the map in which the atomic model was built, giving rise to final estimates of a helical rise of 18.5 Å and a helical twist of 52.9° and a helical rise of 16.4 Å and a helical twist of 55.1°.

RAD51AP1 C59 bound to RAD51 filament formed with Mg^{2+}-ATP

3233 micrographs were imported into cryoSPARC. CTF was estimated using PatchCTF and 812 micrographs were rejected based on poor CTF fit resolution worse than 10 Å and defocus lower than 0.3 µm. 7.5 million particles were picked using a combination of template free filament tracer and template picker and template based filament tracer using 2D averages of a RAD51 filament from a previous screening dataset as templates. After removing overlapping particles, 6.1 million particles were extracted in a small box (180 pixels) and after 2 rounds of 2D classification 1.9 million particles were selected and re-extracted in a larger box (256 pixels), binned 2 times. After further 2D classification, 1.2 million particles were selected and refined with helical refinement, using a reconstruction of the RAD51AP1 bound Ca^{2+}-ATP RAD51 filament as an initial volume and initial helical twist estimate of 56° and a rise estimate of 16 Å. Refinement of the helical parameters during helical refinement led to revised estimates of 55.3° and 16.5 Å respectively. The particles were classified in 3D with a small mask around the density outside of the filament on the central protomer. Two classes with the clearest additional density were combined, and the particles were re-extracted without binning, refined using homogeneous refinement, local CTF refined, and classified in 2D again. The resulting volume was again subjected to 3D classification with a mask around the central RAD51 protomer. Each class was refined using homogeneous refinement and the highest resolution class was selected and classified in 2D again, resulting 97,029 particles. Homogeneous refinement with minimizing per-particle scale and per-particle defocus resulted in a final volume at a resolution of 3 Å. Final helical parameters were estimated based on the central part of the map in which the atomic model was built, giving rise to final estimates of a helical rise of 15.9 Å and a helical twist of 55.8°.

1. I. Brouwer *et al.*, Two distinct conformational states define the interaction of human RAD51-ATP with single-stranded DNA. *EMBO J* **37** (2018).

2. F. Paoletti *et al.*, Molecular flexibility of DNA as a key determinant of RAD51 recruitment. *EMBO J* **39**, e103002 (2020).
3. J. Schindelin *et al.*, Fiji: an open-source platform for biological-image analysis. *Nat Methods* **9**, 676-682 (2012).
4. S. R. Martin, A. Ramos, L. Masino, Biolayer Interferometry: Protein-RNA Interactions. *Methods Mol Biol* **2263**, 351-368 (2021).
5. J. Zivanov, T. Nakane, S. H. W. Scheres, A Bayesian approach to beam-induced motion correction in cryo-EM single-particle analysis. *IUCrJ* **6**, 5-17 (2019).
6. A. Punjani, J. L. Rubinstein, D. J. Fleet, M. A. Brubaker, cryoSPARC: algorithms for rapid unsupervised cryo-EM structure determination. *Nat Methods* **14**, 290-296 (2017).
7. J. He, T. Li, S. Y. Huang, Improvement of cryo-EM maps by simultaneous local and non-local deep learning. *Nat Commun* **14**, 3217 (2023).

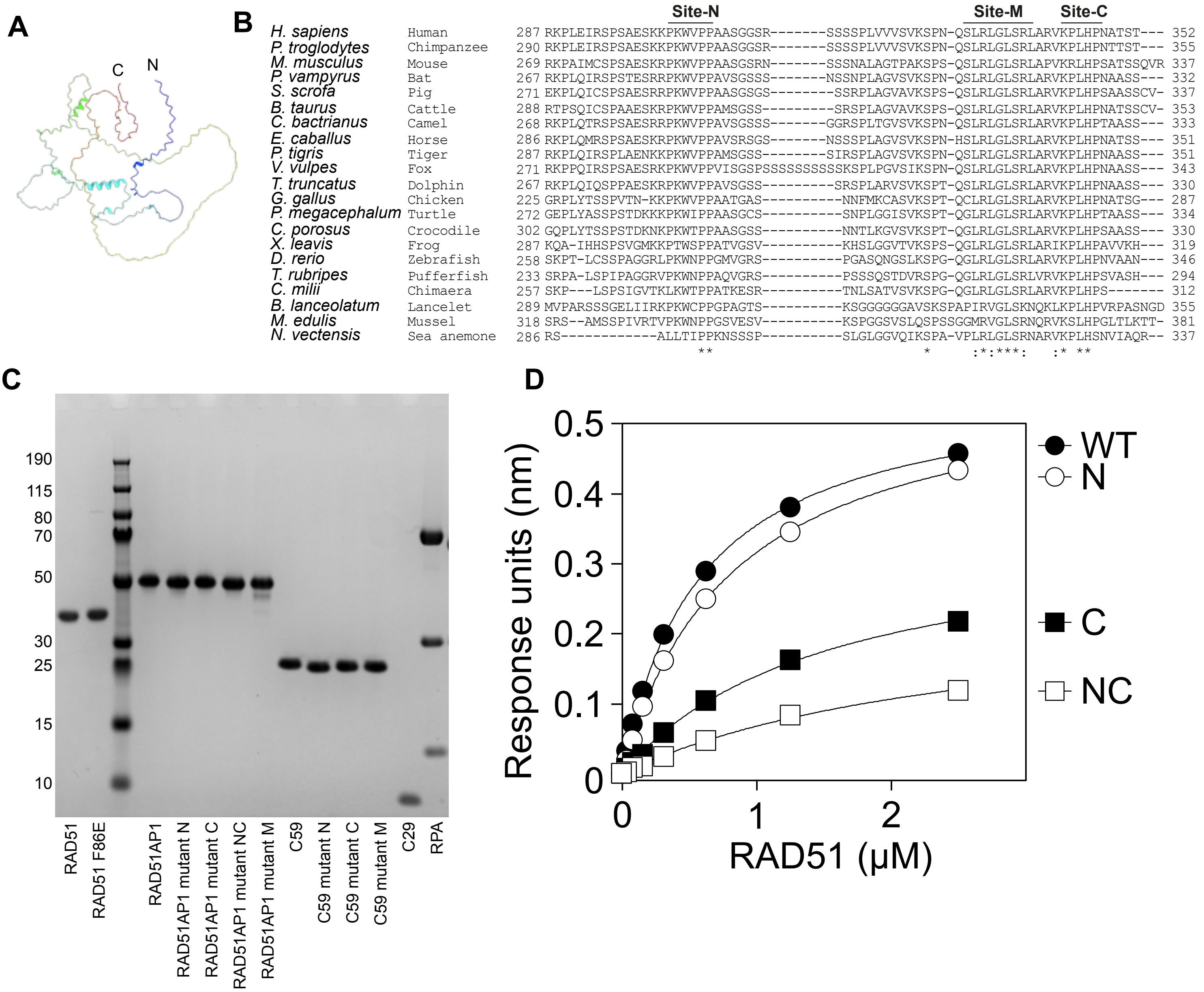

Figure S1. **(A)** Structural prediction by AlphaFold3 showing that RAD51AP1 is largely an intrinsically disordered protein. **(B)** Sequence alignment of the C-terminal region of RAD51AP1 orthologues from indicated species, conserved regions indicated above. **(C)** SDS-PAGE analysis demonstrating the purity of proteins used in this study. **(D)** Binding curves of RAD51 to full-length RAD51AP1 (wild type or mutants) obtained from bio-layer interferometry.

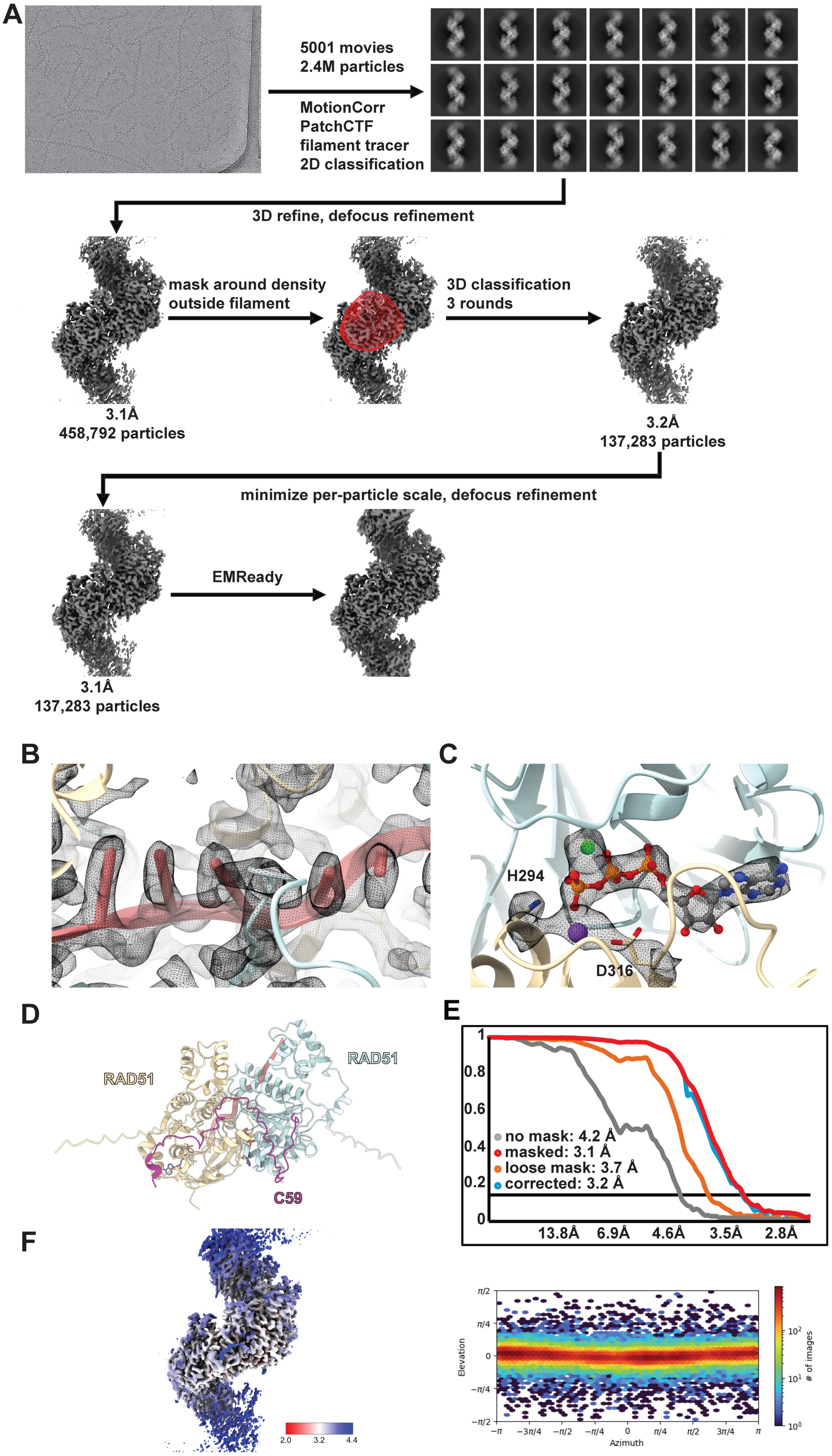

Figure S2. (A) Data processing flowchart of the Ca^{2+}-ATP RAD51 filament bound to full-length RAD51AP1. **(B)** Density corresponding to the DNA. **(C)** Density corresponding to ATP. Green sphere indicates the Mg^{2+}/Ca^{2+}-ATP**.** Purple sphere indicates additional ion bound. **(D)** AlphaFold3 model RAD51 C59 bound to a RAD51 dimer and ssDNA. **(E)** Fourier Shell Correlation (FSC) curves and resolution as determined by FSC=0.143. (F) CryoEM map of the filament from Figure 2I colored by local resolution and orientation distribution of the Ca^{2+}-ATP filament bound by RAD51AP1.

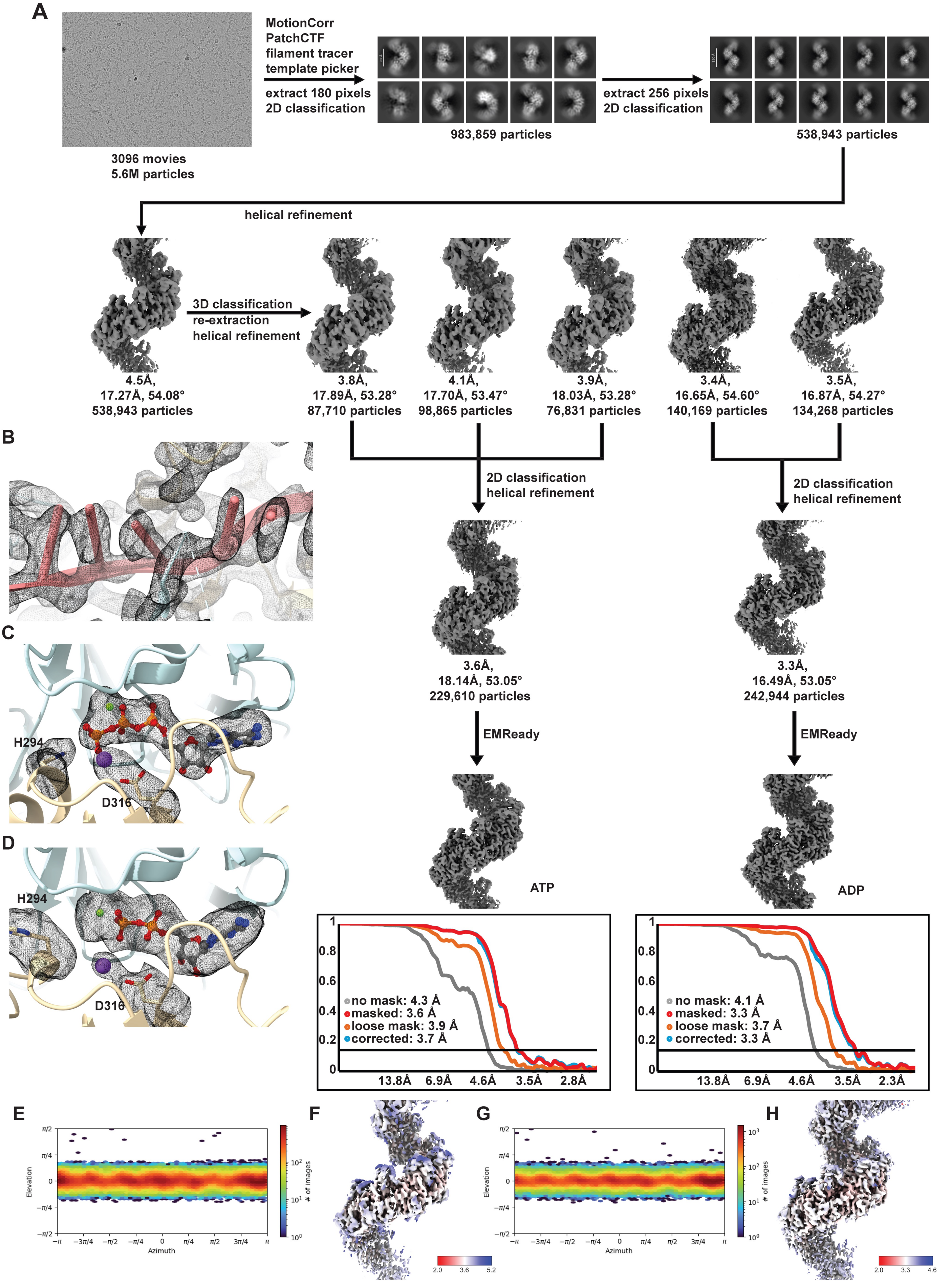

Figure S3. (A) Data processing flowchart of the RAD51-ssDNA filaments in the presence of Mg^{2+}-ATP and Mg^{2+}-ADP including FSC curves. **(B)** Density corresponding to the DNA in the ATP filament. **(C)** Density corresponding to ATP in the ATP filament. **(D)** Density corresponding to ADP in the ADP filament. **(E)** Orientation distribution of the ADP filament. **(F)** CryoEM map of the ADP filament colored by local resolution. **(G)** Orientation distribution of the ATP filament. **(H)** CryoEM map of the ATP filament colored by local resolution.

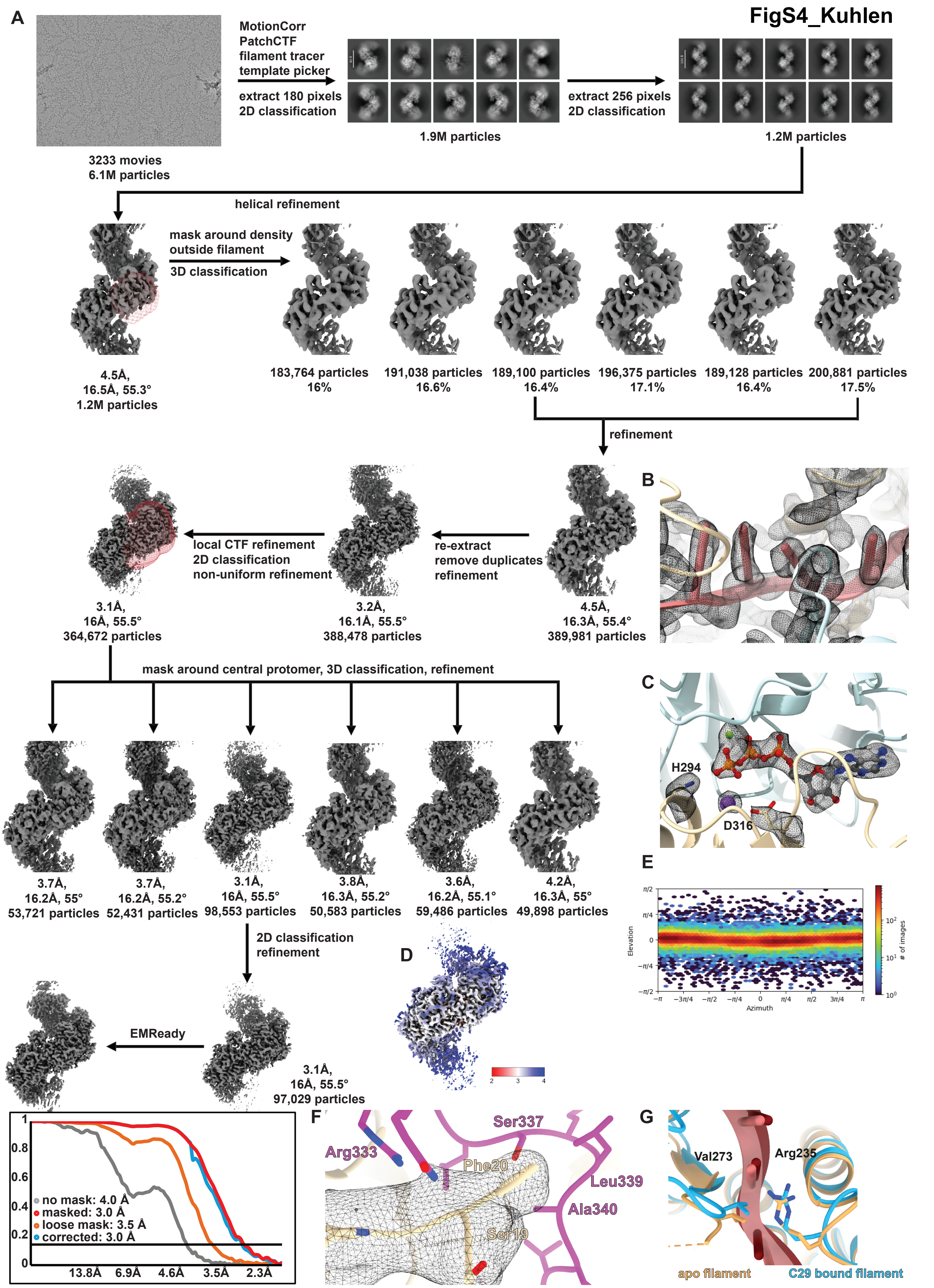

Figure S4. (A) Data processing flowchart of the Mg^{2+}-ATP RAD51 filament bound by C29 including FSC curves. **(B)** Density corresponding to the DNA in the filament. **(C)** Density corresponding to ATP. **(D)** CryoEM map of the filament colored by local resolution. **(E)** Orientation distribution of the filament. (F) Visible density for RAD51 N-terminus including Phe20. (G) comparisons of RAD51-ssDNA (orange) and with RAD51AP1 C29 bound (cyan).

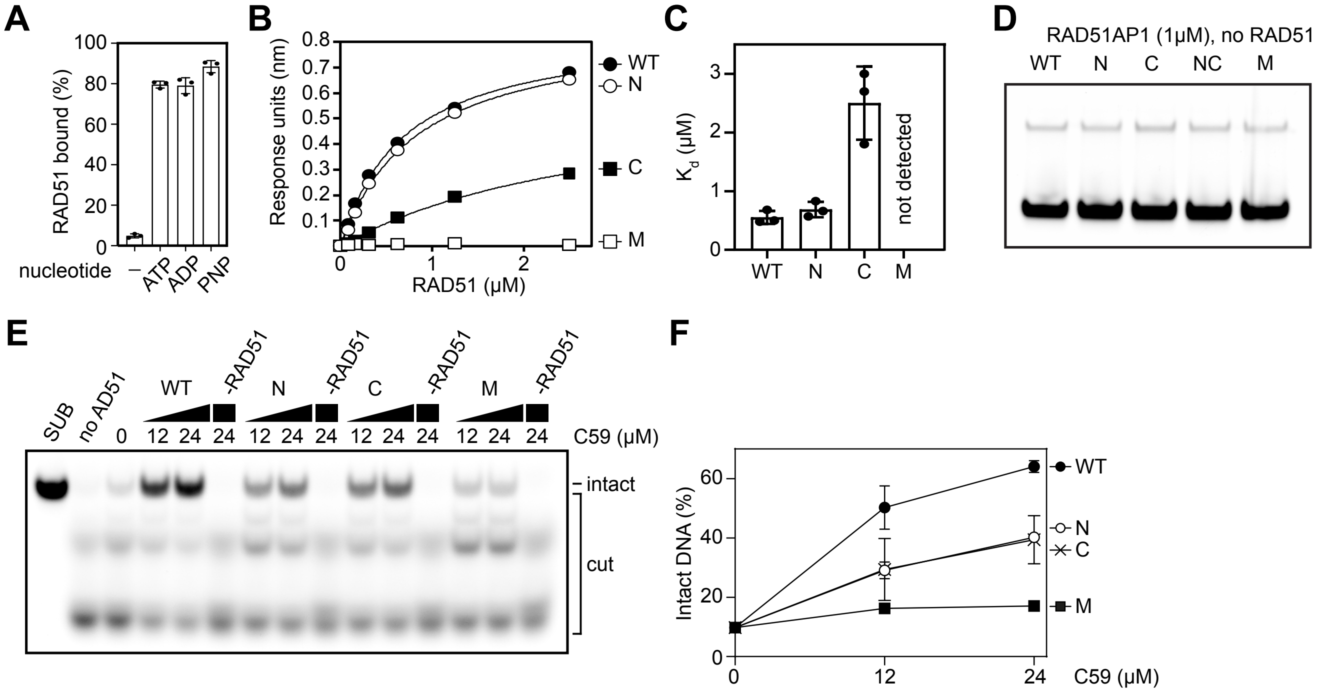

Figure S5. (A) Quantification of Figure 5A. **(B)** Representative binding curves of C59 (wild type or mutants) obtained from bio-layer interferometry. **(C)** K_d values for the binding of C59 and RAD51, determined by bio-layer interferometry. Averages are shown with error bars depicting standard deviation (n = 3). P-values for comparisons of WT with Site-N was 0.9019, WT with Site-C was 0.0017 and Site-N and Site-C was 0.0025. Statistical test was one-way ANOVA followed by Tukey's correction for multiple comparisons. **(D)** Representative gel images showing the stimulation of strand exchange by RAD51AP1 wild-type and mutants at 1 μM in the absence of RAD51. **(E)** Representative gel images showing RAD51 filament stabilization by C59 (wild type or mutants) using the nuclease protection assay. **(F)** Quantification of **(E)**. Averages are shown with error bars depicting standard deviation (n = 3).

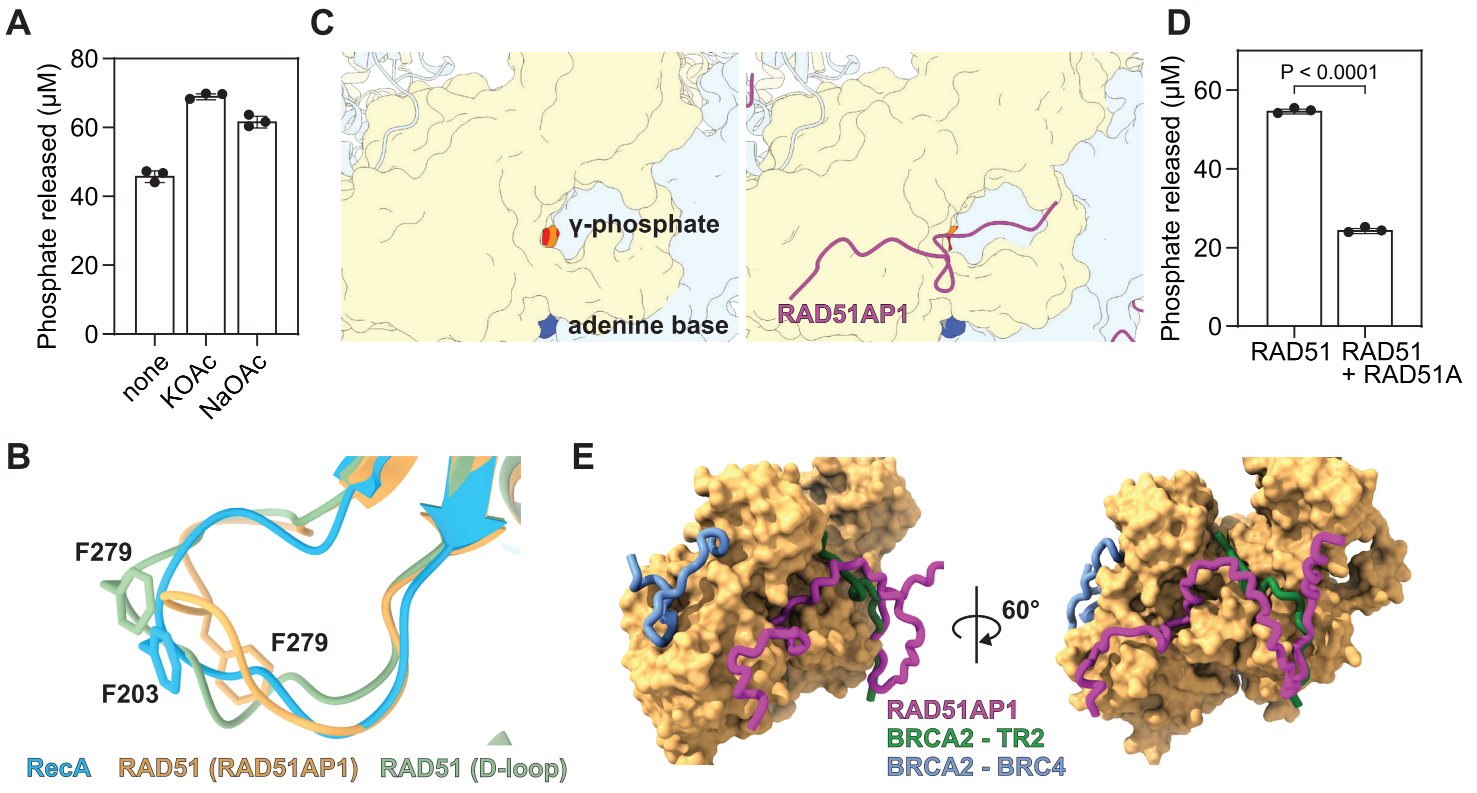

Figure S6. (A) ATPase assay showing the stimulation of RAD51 ATPase activity by 50 mM potassium acetate (KOAc) and sodium acetate (NaOAc). **(B)** View of the L2 loop in an overlay of the structure of the RAD51 (9I62) and RecA (7IY7) filaments during homologous recombination (D-loop) and the structure of the RAD51 filament bound by C29. **(C)** View of a possible release channel for the γ-phosphate in the Mg^{2+}-ATP filament (left) and the Mg^{2+}-ATP filament bound by RAD51AP1 C29 (right). **(D)** RAD51 ATPase activity in the presence of RAD51AP1. Statistical test was an unpaired T test. **(E)** Overylay of the structures of a RAD51 dimer bound by BRCA2 TR2 (8uvw), C59 (as in Figure 2I) and BRCA2 BRC4 (1n0w).
